# Integrative Single-Cell and Spatial Transcriptomic Analysis Identifies a Tertiary Lymphoid Structure-Associated LAMP3^+^CCR7^+^ mregDC Antigen-Presentation Program in Ovarian Cancer

**DOI:** 10.3390/cancers18142259

**Published:** 2026-07-14

**Authors:** Feifan Lu, Ting Zhang, Zhixuan Li, Renqi Yao, Hao Hu, Rui Guan, Mingjuan Xu

**Affiliations:** 1 Department of Obstetrics and Gynecology, Changhai Hospital, Naval Medical University, Shanghai 200433, China; luff94@163.com; 2 Department of Hematology, Shanghai East Hospital, Tongji University, Shanghai 200120, China; 13291028995@163.com; 3 National Facility for Translational Medicine (Beijing), Medical Innovation Research Division, PLA General Hospital, Beijing 100853, China; leezx7925@163.com (Z.L.); yaorenqixx1995@163.com (R.Y.); 4 Department of Pathology, Changhai Hospital, Naval Medical University, Shanghai 200433, China; hh_chpathology@163.com

**Keywords:** ovarian cancer, tertiary lymphoid structure, mregDC, spatial transcriptomics, single-cell RNA sequencing, antigen presentation, immune infiltration, translational immuno-oncology

## Abstract

**Background:** Tertiary lymphoid structures (TLSs) are spatial immune niches in solid tumors, but their relationship to mature regulatory dendritic-cell (mregDC) states in ovarian cancer remains incompletely resolved. **Methods:** We integrated public gynecological-tumor single-cell RNA sequencing, representative CD11C/HLA-DRA/LAMP3/CCR7 multiplex immunofluorescence, public Xenium and multi-sample spatial transcriptomics, TCGA-OV immune deconvolution and exploratory prognostic modeling, scTenifoldKnk and CellOracle perturbation analyses, and supplementary computational drug prioritization. **Results:** A $LAMP3^{+}CCR7^{+}$ dendritic-cell state showed mature migratory, antigen-presentation, checkpoint, and NF-κB/TNF-associated programs. Spatial analyses linked TLS-score-defined regions to mregDC, antigen-presentation/MHC-II proxy, and interferon-associated signals, with distance-gradient analyses supporting TLS-proximal enrichment. Immune deconvolution associated the TLS/mregDC axis with an immune-infiltrated TCGA-OV contexture, whereas adjusted analyses emphasized dependence on broader immune-program richness. Perturbation analyses nominated candidate regulatory axes for experimental testing, and the supplementary drug-prioritization layer was retained only as target-class hypothesis support. **Conclusions:** These data support a hypothesis-generating model of a TLS-associated $LAMP3^{+}CCR7^{+}$ mregDC antigen-presentation program in ovarian cancer, while requiring raw-channel tissue validation, functional perturbation, and independent clinical testing.

## Simple Summary

Ovarian cancer often contains immune-rich regions, but the spatial relationship between tertiary lymphoid structures (TLSs) and dendritic-cell states remains incompletely defined. We integrated public single-cell and spatial transcriptomic datasets, representative four-marker immunofluorescence, patient-level immune deconvolution, exploratory survival modeling, and computational perturbation analyses. The results support a cautious model in which $LAMP3^{+}CCR7^{+}$ mature regulatory dendritic-cell programs are enriched in or near TLS-score-defined immune fields and are associated with antigen-presentation/MHC-II proxy, interferon, and patient immune-context features. This study does not claim causal function or clinical treatment efficacy; instead, it provides a transparent framework for prioritizing TLS-associated mregDC programs for future raw-channel pathology validation and immunotherapy-oriented experimental testing.

## 1. Introduction

Gynecological malignancies remain a major challenge to women’s health, and ovarian cancer (OVC) is particularly difficult to manage once advanced. Although immune checkpoint blockade has improved outcomes in selected gynecological cancers, its benefit in OVC has generally been limited because robust predictive biomarkers are lacking and the tumor microenvironment is highly heterogeneous [1,2,3,4,5,6,7]. A clearer definition of the immune architecture of OVC may therefore help explain why some tumors appear inflamed yet remain therapeutically resistant.

Dendritic cells (DCs) are central antigen-presenting cells, but tumor-associated DC states may contribute to either immune activation or immune regulation [8,9,10]. Across tumor types, single-cell studies have described mature LAMP3/CCR7-enriched DC programs that are variably termed mregDCs, DC3-like cells, or $CCR7^{+}LAMP3^{+}$ states [11,12,13]. A recent pan-cancer single-cell analysis further refined tumor-infiltrating DC subsets and emphasized tissue-specific DC programs, reinforcing the need to interpret mregDCs as context-dependent cell states rather than as a fixed lineage [14]. Because the transcriptional boundaries and functional roles of these states vary across tissues, we use the term mregDCs descriptively for the OVC-associated $LAMP3^{+}CCR7^{+}$ dendritic-cell state while providing representative tissue-image support with a DC/APC-focused immunofluorescence panel. Tertiary lymphoid structures (TLSs) have likewise been linked to local immune activation and immunotherapy relevance in multiple tumors [15,16]. Recent TLS studies have moved the field from binary TLS detection toward spatial location, maturation state, and composite TLS metrics, including ovarian-cancer-specific immunotherapy perspectives and pan-cancer spatial TLS atlases [17,18,19]. Importantly, the pan-cancer spatial atlas reported limited TLS detection in the OVCA spatial-transcriptomic subset while identifying abundant segmented TLSs in an HGSOC H&E therapy cohort, underscoring that ovarian TLS biology should be framed around TLS-positive regions and maturation composition rather than disease-wide TLS abundance [17]. However, whether mregDC states are spatially associated with TLS programs in OVC remains unclear.

Recent work has also emphasized that single-cell biomarker discovery increasingly requires patient-level feature construction, multigene modeling, and downstream biological interpretation rather than simple one-marker differential-expression screening [20]. In parallel, in silico perturbation methods such as scTenifoldKnk and CellOracle provide a practical framework for prioritizing regulatory nodes from single-cell gene-regulatory networks when experimental perturbation is not immediately feasible [21,22]. These approaches do not replace functional validation, but they can help connect cell-state markers, spatial localization, and candidate regulatory programs into a testable hierarchy.

The specific aim of this study was to determine whether OVC contains a recurrent $LAMP3^{+}CCR7^{+}$ dendritic-cell state, whether this state can be visualized in representative tissue fields with a focused CD11C/HLA-DRA/LAMP3/CCR7 marker panel, and whether operational TLS-score-defined domains contain spatially coordinated mregDC and antigen-presentation proxy programs. To address this aim, we integrated scRNA-seq, multiplex immunofluorescence, Xenium spatial transcriptomics, multi-sample spatial transcriptomic support, TCGA/GTEx-based bulk analyses, patient-level composite modeling, exploratory in silico perturbation analyses, and a supplementary drug-prioritization layer. Consistent with the primarily translational and computational design of the study, these analyses were intended to assemble transcriptional, spatial, immune-context, prognostic, and perturbation-prioritization evidence rather than claim definitive causality, temporal TLS maturation, niche specificity beyond immune richness, or clinical drug efficacy. Accordingly, spatial antigen-presentation signals are reported as MHC-II/AP proxies where direct HLA-family genes are unavailable; the four-marker immunofluorescence layer is interpreted as representative tissue-image support; and all prognostic, perturbation, and supplementary drug analyses are exploratory. The downstream patient-level, virtual-perturbation, and drug-prioritization analyses were used to define testable hypotheses about immune contexture and regulatory nodes, not to establish a clinically deployable biomarker or treatment strategy.

## 2. Materials and Methods

### 2.1. Study Design and Data Sources

All analyses were conducted using public datasets or ethically approved clinical specimens and were designed as a descriptive, multimodal assessment of the OVC immune microenvironment. Because the public single-cell, spatial-transcriptomic, bulk-transcriptomic, and clinical datasets are static snapshots, the study was not designed to infer temporal tumor evolution, dynamic TLS maturation, or treatment-induced immune remodeling. The GEO scRNA-seq cohort comprised 22 gynecological tumor samples assembled from three public accessions: GSE197461 and GSE208653 for the cervical component, including 5 cervical squamous cell carcinoma (CESC/CSCC) and 6 cervical adenocarcinoma (CEAD/ADC) samples, and GSE173682 for the ovarian and endometrial component, including 5 OVC and 6 uterine corpus endometrial carcinoma/endometrial cancer (UCEC/EC) samples [23,24,25]. The accession-level sample list and disease/sample-count summary are provided in Supplementary Table S1. This integrated scRNA-seq cohort was used as a pan-gynecologic immune reference for recurrence of the mregDC state, not as an organ-matched comparative cohort; organ-of-origin differences are therefore inseparable from source-study and platform effects in this integrated atlas. Multiplex immunofluorescence was performed on FFPE sections following routine pathological review. Spatial analyses were performed on one public FFPE OVC Xenium Prime dataset containing 407,124 cells generated with the Xenium Human 5K panel plus 100 custom targets [26,27,28]. Additional marker-workflow and perturbation analyses used the public 10x Genomics 17k human ovarian cancer scFFPE matrix containing 17,553 cells. Additional multi-sample spatial and 10x dataset accessions are listed in Supplementary Table S3. TCGA-OV STAR-count RNA-seq and clinical data were used for immune-correlation and retrospective survival analyses, and GTEx normal-tissue data were used where normal reference comparisons were required [29].

### 2.2. Single-Cell Transcriptomic Analysis

Raw FASTQ files were aligned to the GRCh38 reference genome with Cell Ranger (v6.1.2), and count matrices were processed in Seurat (v4.3.0) [30,31,32]. Cells with fewer than 300 genes, more than 6000 genes, or more than 10% mitochondrial transcripts were excluded [33,34]. After normalization and Harmony-based batch correction, 31,757 high-quality cells were retained. Highly variable genes were identified, the top 30 principal components were used for dimensionality reduction, and graph-based clustering was performed at a resolution of 0.8 [35,36,37,38,39]. Major immune populations were annotated by differential marker analysis (FindAllMarkers, Wilcoxon test) with reference to published pan-cancer myeloid atlases and the CellMarker database [40,41].

Cell-type composition within the immune-cell dataset was summarized with stacked bar plots, box plots, sample-level heatmaps, and correlation matrices [42,43]. Because the single-cell dataset was immune-cell focused, these summaries were interpreted as relative immune-state composition rather than unbiased whole-tissue cellular abundance. Potential ligand–receptor interactions were analyzed with CellChat (R package; the archived analysis record did not retain the minor release number) using CellChatDB.human and were interpreted as candidate communication axes rather than direct functional evidence [44]. Pseudotime analysis was performed using Monocle 2 (R package; the archived analysis record did not retain the minor release number) and focused on dendritic-cell states; T/NK cells shown in the trajectory figure were retained for visual context but not for lineage inference [45]. In light of prior reports that canonical cDC1 and cDC2 states can acquire a $LAMP3^{+}$ mature regulatory/migratory DC program after tumor-antigen exposure, pseudotime was interpreted as a state-relationship analysis rather than evidence of a linear cDC2-to-cDC1 transition or definitive lineage conversion [11,13,40,46]. Functional scores for dendritic-cell and T/NK programs were calculated with AddModuleScore, and differentially expressed genes in cDC-M09-LAMP3 were analyzed with DESeq2, followed by GO/KEGG enrichment using clusterProfiler [47,48,49].

### 2.3. Pathology, Multiplex Immunofluorescence, and Spatial Analysis

Hospital specimens underwent routine H&E evaluation and diagnosis according to standard histopathological criteria for female genital tumors [50]. Multiplex immunofluorescence was designed to provide representative tissue-image support for pan-gynecologic mregDC-associated staining using a four-marker panel comprising CD11C, HLA-DRA, LAMP3, and CCR7. CD11C and HLA-DRA were used to mark APC-enriched regions, whereas LAMP3 and CCR7 were used to identify mature migratory mregDC-associated staining in immune-enriched regions. Standard TSA-based staining, DAPI counterstaining, and multispectral imaging were performed. Representative-image review was performed for one case per cancer type (OVC, CEAD, CESC, and UCEC) and one selected ROI per case using flattened merged JPGs. APC-like events and regional four-channel co-occurrence events were identified using predefined merged-RGB local color thresholds. For each selected crop, the image-derived event density was calculated as the corresponding event count divided by the displayed crop area in mm^2^, and the regional four-channel co-occurrence fraction was calculated as the number of regional four-channel events divided by the number of CD11C/HLA-DRA APC-like events. These values are descriptive selected-crop image metrics only; they are not case-level whole-tissue densities, cell-resolved four-marker positivity, Pearson or Manders co-localization coefficients, or cross-cancer abundance estimates. Marker co-occurrence and regional enrichment were interpreted together with the transcriptomic evidence rather than as stand-alone proof of function.

For Xenium data, signals were processed with Xenium Onboard Analysis v3.0.0, Xenium Explorer v3.2, and Xenium Ranger v3.1 (10x Genomics, Pleasanton, CA, USA). Spatial interpretation focused on mregDC-associated signals and TLS-score-defined regions in OVC. Because exact HLA-DRA and HLA-family genes were absent from both the public Xenium panel and the public 10x scFFPE matrix used here, public transcriptomic analyses treated CD74, B2M, TAP1, TAP2, CIITA, NLRC5, PSMB8/9, and related antigen-presentation genes as antigen-presentation/MHC-II proxy signals when available. Direct HLA-DRA evidence was assigned to the immunofluorescence panel. TLS regions were operationally defined by concordant layers: a 12-chemokine TLS module; a patient-level 29-gene TLS imprint; an expression-overlap spatial TLS imprint where full spatial gene coverage was not available; and spatial co-localization with B-cell follicle, T-cell zone, Tfh/germinal-center, antigen-presentation, and mregDC-associated scores. Full signature definitions, legacy-label harmonization, and per-dataset gene-coverage audits are provided in Supplementary Table S2 and source_data/Tables_signature_and_GEO. Spatial density contours, co-high enrichment, k-nearest-neighbor neighborhood fractions, Moran’s I, TLS-ranked decile analyses, and distance-to-TLS-field bins were then used to test whether mregDC-associated signals localized within or adjacent to these TLS-score-defined domains and whether TLS-proximal fields showed coordinated antigen-presentation/interferon programs. The Fig3 CD11C/HLA-DRA/LAMP3/CCR7 immunofluorescence panel was used as representative tissue-image support for the presence of mregDC-associated staining across gynecological tumor types and was not used by itself to define TLS identity. As supplementary spatial feature analyses, a deterministic 60,000-cell subset was sampled from the public Xenium section with matched x/y coordinates and 1500 real Xenium genes. singleCellHaystack 1.0.3 was used to rank spatially non-random features, and Hotspot 1.1.3 was used to compute spatial autocorrelation on an h5ad object generated from the same subset with the count layer and spatial coordinates. The corresponding singleCellHaystack and Hotspot audit outputs are shown in Supplementary Figures S1 and S2. The Hotspot local-correlation/module step was not used for interpretation because 1269 FDR-significant genes made pairwise module computation impractical in the current run. These analyses were used as spatial non-randomness, autocorrelation, and module-support audits rather than as histological TLS-maturation staging.

### 2.4. Bulk Immune Infiltration, Prognostic Modeling, and Virtual Perturbation Analyses

TCGA STAR counts were converted to TPM and log^2^(TPM+1)-transformed. Immune infiltration was estimated with a ten-method human tumor microenvironment deconvolution framework implemented through IOBR v2.2.1 and immunedeconv v2.1.4: quanTIseq, TIMER, CIBERSORT, CIBERSORT-ABS, MCP-counter, xCell, EPIC, ESTIMATE, ABIS, and ConsensusTME [51,52]. TIMER and ABIS were run through direct immunedeconv functions after the generic wrapper failed for these two methods; TIMER used ovarian cancer as the indication for all TCGA-OV profiles, and ABIS was run on the same TPM patient-level expression matrix. TLS/mregDC patient-level scores were correlated with method-specific immune-cell estimates and collapsed into immune-class summaries only after retaining method provenance. LAMP3 was first screened across gynecological tumors using GEPIA, after which OVC was selected for downstream retrospective prognostic analyses [29].

Patients were stratified primarily by median and quartile LAMP3 expression; data-derived optimal-cutoff analysis (surv_cutpoint in survminer) was treated as a sensitivity analysis because of the risk of optimistic separation without external validation. For patient-level modeling, mregDC-associated features, lymphoid/TLS-associated features, immune-deconvolution scores, and selected clinical covariates were integrated as candidate predictors. Lasso regression with 10-fold cross-validation (glmnet; seed 20260528) used the lambda.min rule for coefficient selection, followed by Cox proportional hazards modeling, stepwise model selection, Kaplan–Meier analysis, time-dependent ROC analysis, calibration-by-risk-quintile analysis, decision-curve analysis (ggDCA), and nomogram construction (rms) to evaluate exploratory prognostic performance rather than derive a clinically validated predictor [20,53,54,55,56,57]. For clinical sensitivity analysis, GDC TCGA-OV clinical fields were matched to the model cohort, and a Cox model was adjusted for age, advanced FIGO stage (III/IV vs. I/II), and high grade (G3/G4/GB vs. G1/G2). The adjusted analysis included 416 patients after clinical-covariate complete-case filtering from the 426-patient model cohort. Residual disease was audited but not included because only 14 of 426 model patients had a recorded value.

To address whether mregDC/TLS/AP coupling reflected generic immune-hot tumors, we performed immune-richness-adjusted specificity analyses. At the TCGA-OV patient level, immune estimates were collapsed by patient after retaining method provenance, and composite controls were constructed for broad immune context, tumor purity, B-lineage scores, T-lineage scores, and a myeloid/APC sensitivity control. Partial Spearman correlations were calculated by rank-transforming variables, residualizing both variables of interest against the control matrix with ordinary least-squares regression, and correlating the residuals. Spatial spot-level analyses used the multi-sample spatial downsample table and controlled mregDC–TLS associations for antigen-presentation/MHC-II, B-cell/TLS, and T-cell-zone scores; the reciprocal mregDC–antigen-presentation analysis controlled for TLS, B-cell/TLS, and T-cell-zone scores. Xenium cell-level sensitivity analyses controlled for antigen-presentation proxy, interferon-response, tumor-proliferation, and tumor-EMT/invasion programs. These adjusted tests were interpreted as confounding audits, not as proof of causal niche specificity.

For virtual perturbation analysis, scTenifoldKnk v1.0.3 virtual knockout was performed on 6000 sampled cells from the 10x scFFPE matrix using 858 high-variable or marker-retained genes. ITGAX, LAMP3, CCR7, and CD74 were tested as marker/proxy knockout targets, with CD74 used as the antigen-presentation proxy because HLA-DRA and HLA-family genes were absent from the public matrix [21]. CellOracle 0.20.0 transcription-factor perturbation was then performed in Ubuntu-22.04 WSL using the human hg38 promoter base GRN hg38_gimmemotifsv5_fpr2, 5000 sampled cells, 2219 retained genes, and 149 expression-overlapping transcription factors [22]. Candidate perturbed transcription factors included RELA, RELB, NFKB1, NFKB2, STAT1, STAT3, IRF1, IRF4, IRF8, BATF3, SPI1, and CEBPB. CellOracle results were interpreted as transcription-factor-level perturbations of upstream regulators, not as direct knockout of non-transcription-factor markers such as LAMP3 or CCR7. These analyses were used for hypothesis prioritization only and were not interpreted as experimental knockout evidence.

For the drug-prioritization layer, regulators prioritized by TLS/mregDC perturbation analyses were mapped to targetable classes, including JAK, IKK/NF-κB, proteasome, polyphenol, and STAT3-inhibitor classes. Candidate compounds were ranked by a composite virtual-screening score incorporating regulator mapping, predicted TLS/mregDC program reversal, high-risk signature reversal, and available clinical/target evidence. This drug layer did not use cell-line IC50 measurements, PIK3CA mutation annotations, or pharmacogenomic response modeling; it therefore should not be interpreted as a PIK3CA-directed drug-sensitivity analysis. A molecular-docking audit was performed for receptor-ligand pairs with available co-crystal or receptor structures using Open Babel v3.1.1 for ligand/receptor preparation and AutoDock Vina v1.2.7 for non-covalent redocking. Three pairs completed Vina scoring, whereas two boronate proteasome-inhibitor pairs were marked Vina-ineligible because standard non-covalent Vina atom typing does not adequately represent boronate covalent chemistry. This analysis is provided as Supplementary Figure S7 and was interpreted as drug prioritization and docking feasibility, not as evidence of therapeutic efficacy.

### 2.5. Statistics

Statistical analyses were performed in R v4.5.3. Unless otherwise specified, group comparisons used non-parametric tests, correlations used Spearman coefficients, and statistical significance was defined as p<0.05 [58,59,60].

## 3. Results

### 3.1. A Multimodal Cross-Tumor Framework Identifies the OVC Immune Context for TLS-Associated mregDC Analysis

**Figure 1:**
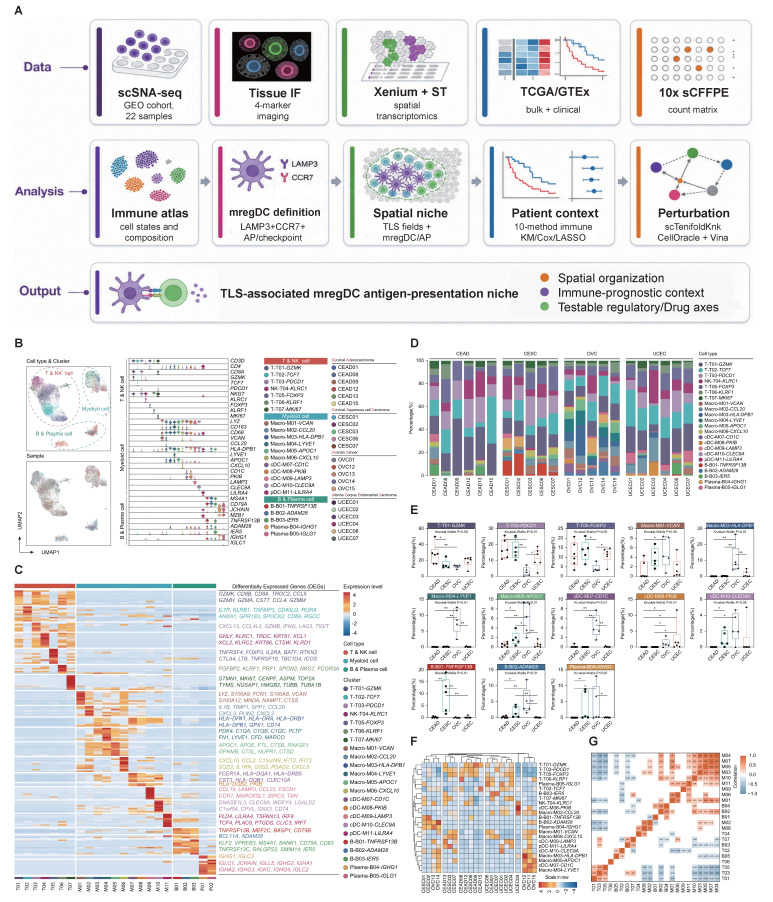
Study design and cross-tumor single-cell immune reference. (**A**) Overview of the multimodal workflow integrating public scRNA-seq, four-marker IF, spatial transcriptomics, patient-level modeling, virtual perturbation, and supplementary drug-prioritization analyses; colors distinguish the data and analysis layers shown in the panel. (**B**) Integrated immune-cell atlas and marker-supported annotation of the gynecological tumor scRNA-seq cohort; each point represents one cell, and colors denote annotated immune states or sample groups as indicated. (**C**) Differentially expressed marker genes defining major immune subsets and refined cell states. (**D**) Sample-level immune-cell composition across CEAD, CESC, OVC, and UCEC specimens; stacked-bar colors denote annotated cell states. (**E**) Cross-tumor comparisons of selected immune-cell-state proportions; points are samples, boxes show the median and interquartile range, and asterisks denote Wilcoxon-test significance (* p<0.05, and ** p<0.01). (**F**) Heatmap summary of sample-level immune-state composition. (**G**) Correlation structure among annotated immune-cell states, with red and blue denoting positive and negative correlations, respectively. The cross-tumor panels provide immune-reference context and should not be interpreted as organ-matched statistical comparisons of basal mregDC abundance.

The final analytical framework combined public scRNA-seq, tissue immunofluorescence, Xenium spatial transcriptomics, multi-sample spatial validation, TCGA/GTEx patient-level analyses, virtual perturbation, and a supplementary drug-prioritization layer (Figure 1). The objective of this first layer was to define the immune-cell reference context for the later OVC-focused spatial analyses, not to compare cervical, ovarian, and endometrial tumor microenvironments as organ-matched cohorts. The scRNA-seq reference comprised 22 gynecological tumor specimens assembled from GSE197461, GSE208653, and GSE173682, including CEAD, CESC, OVC, and UCEC. After quality control and integration, 31,757 cells were resolved into 23 immune subpopulations spanning T/NK, myeloid, dendritic-cell, B-cell, and plasma-cell states. OVC showed a relatively myeloid-rich immune composition in this immune-cell-focused dataset, with higher macrophage and dendritic-cell representation and lower exhausted/regulatory T-cell representation than several comparator gynecological tumor groups. These composition summaries were used to define immune-state context rather than unbiased whole-tissue cellular abundance. Because disease group, organ of origin, accession, and source-study effects are partially confounded in this integrated atlas, cross-organ differences in basal mregDC magnitude were treated as descriptive context rather than as a powered organ-specific comparison.

Within the dendritic-cell compartment, a small cDC-M09-LAMP3 population was detected across tumor types and was enriched for LAMP3; CCR7; and additional maturation, migration, antigen-presentation, and checkpoint-related genes. The presence of this state across gynecological tumor groups supported its recurrence in the reference atlas, whereas between-organ abundance differences require larger, organ-balanced cohorts with harmonized sampling. Sample-level immune-program heatmaps and correlation matrices further separated myeloid-enriched programs from exhausted/regulatory T-cell programs, placing the OVC immune context in a framework suitable for downstream spatial and patient-level analyses.

### 3.2. Single-Cell Analyses Define a Mature Migratory mregDC State with Inflammatory and Antigen-Presentation Features

**Figure 2:**
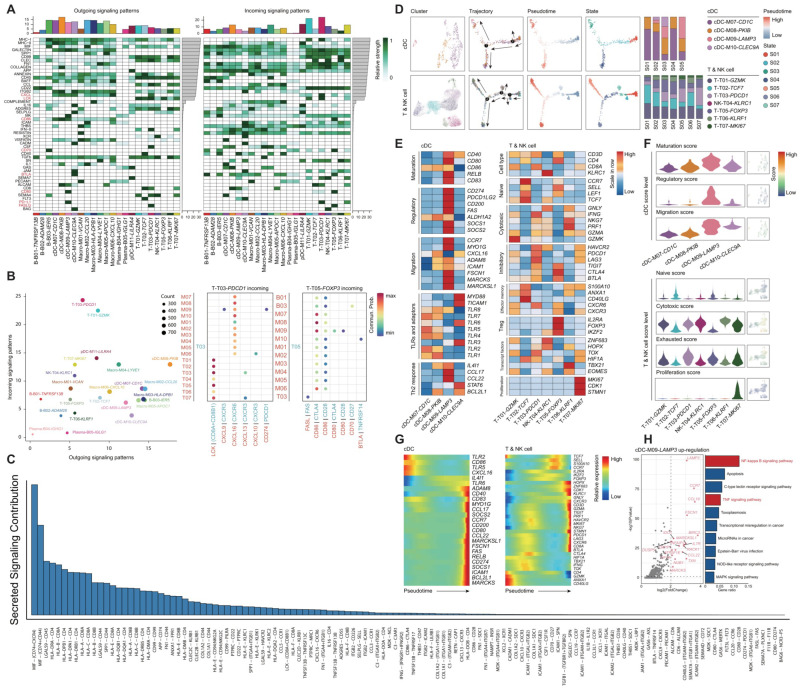
Single-cell analysis defines a mature migratory mregDC program in OVC. (**A**) Outgoing and incoming cell-cell communication patterns across immune-cell states; heatmap intensity and top bars summarize relative signaling strength. (**B**) Signaling-pattern map and representative incoming ligand–receptor interactions involving regulatory/exhausted T-cell states; dot color encodes interaction strength and dot size encodes statistical evidence. (**C**) Ranking of secreted signaling contributions. (**D**) cDC and T/NK-cell state mapping, trajectory structure, pseudotime, and patient-state composition; black trajectory curves and arrows indicate the inferred state path, S01–S07 denote Monocle states, and stacked-bar colors denote annotated subclusters. (**E**) Marker and program heatmaps for cDC and T/NK-cell subclusters. (**F**) Module-score distributions for maturity, regulatory, migration, cytotoxic, exhaustion, and proliferation programs. (**G**) Pseudotime-associated gene-expression dynamics in cDC and T/NK compartments. (**H**) Up-regulated genes and pathway enrichment for the cDC-M09-LAMP3 state; colors denote pathway classes shown in the panel.

Cell-cell communication analysis suggested that myeloid populations were major signal senders and T-cell populations were the predominant receivers, with representative CD86–CTLA4 and CD80–CD28 interactions (Figure 2). In the dendritic-cell compartment, cDC-M09-LAMP3 localized near canonical cDC-M07-CD1C and cDC-M10-CLEC9A states in state-relationship analyses while showing the highest maturity, migration, regulatory, checkpoint, and antigen-presentation scores. This state expressed CD40, CD80, CD86, CD274, PDCD1LG2, CCR7, and MYO1G, consistent with a mature migratory DC phenotype.

Because previous studies show that canonical cDC1 and cDC2 populations can acquire a $LAMP3^{+}$ mregDC program after tumor-antigen exposure, the pseudotime and trajectory-related panels were interpreted as transcriptional state relatedness rather than definitive lineage conversion [11,13,40,46]. Differential-expression and pathway analyses showed upregulation of LAMP3, CCR7, CCL19, and FSCN1 together with enrichment of NF-κB, TNF, and C-type lectin receptor signaling, supporting a mature migratory dendritic-cell state with inflammatory and T-cell-directed features.

### 3.3. Four-Marker Immunofluorescence Provides Representative Pan-Gynecologic Tissue-Image Support for mregDC-Associated Staining

**Figure 3:**
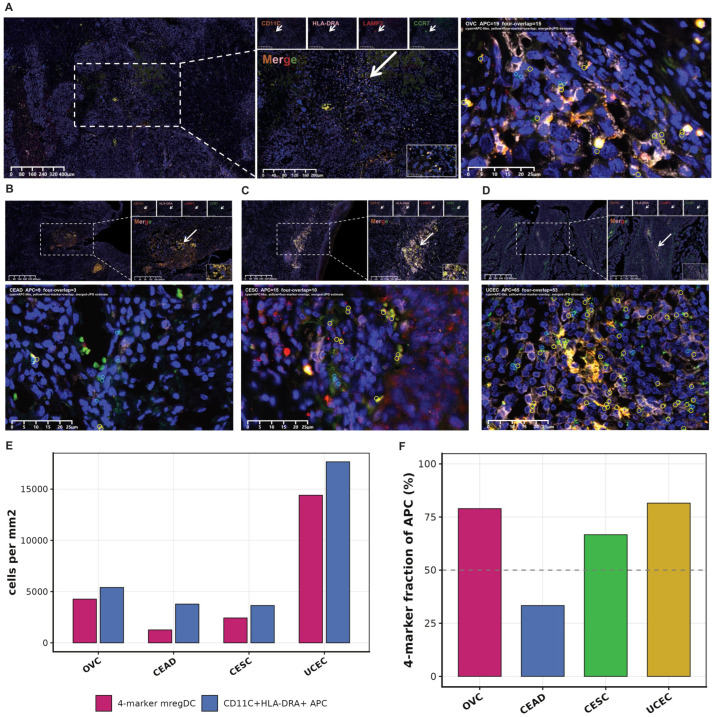
Four-marker multiplex immunofluorescence supports pan-gynecologic mregDC-associated staining. (**A**) Representative OVC field showing CD11C, HLA-DRA, LAMP3, and CCR7 staining from low-magnification tissue context to an enlarged immune-enriched ROI. (**B**–**D**) Representative CEAD, CESC, and UCEC immune-enriched fields and enlarged ROIs, respectively. Dashed boxes and connecting lines identify the enlarged regions, and white arrows indicate marker-enriched foci. In the merged-image review overlays, yellow circles mark CD11C/HLA-DRA APC-like events and cyan circles mark regional four-channel co-occurrence events identified by the predefined merged-image threshold. Scale bars are embedded in each image and labeled in μm; enlarged ROI panels use a 25-μm scale. (**E**) Selected-crop image-derived event densities for CD11C/HLA-DRA APC-like events and regional four-channel events, calculated as raw event count divided by displayed crop area. (**F**) Regional four-channel co-occurrence events expressed as a percentage of APC-like events in the same selected crop. One case and one selected ROI were reviewed per tumor type. Panels (**E**,**F**) are descriptive image-derived metrics from flattened merged images and do not establish case-level whole-tissue density, cell-resolved four-marker positivity, formal Pearson or Manders co-localization, cross-cancer abundance differences, or TLS identity.

**Figure 4:**
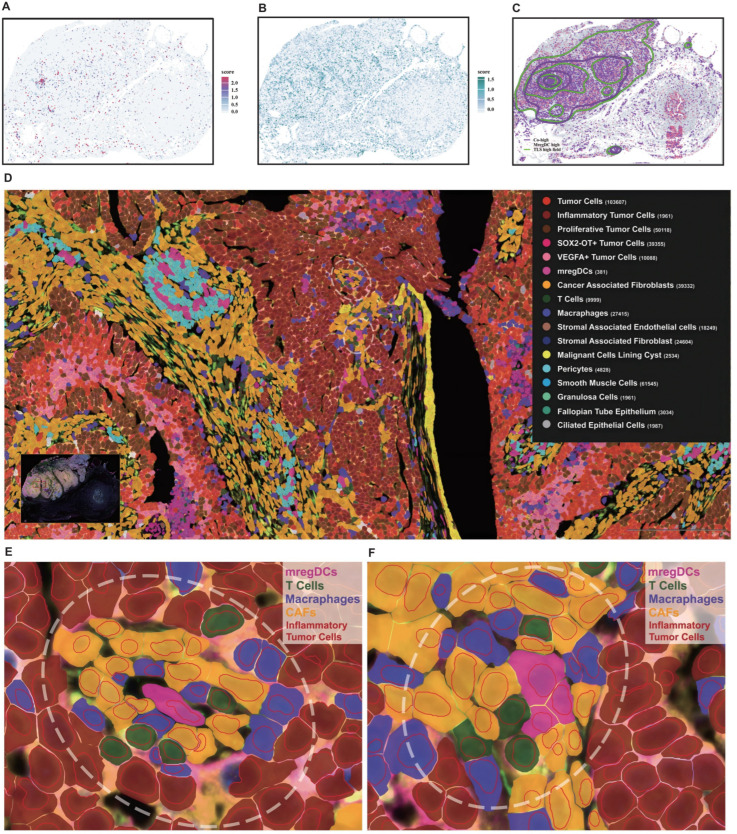
Xenium segmentation localizes mregDC/AP-proxy signals to TLS-score-defined fields. (**A**) Cell-level TLS-program score map in the public OVC Xenium section. (**B**) Cell-level mregDC/AP-proxy score map in the same spatial coordinate frame. Score-map color gradients run from low to high values as shown by the color bars. (**C**) TLS-high and mregDC-high field contours with co-high regions; green and purple contours denote TLS-high and mregDC-high fields, respectively. (**D**) Full-section cell-class segmentation overlaid on the tissue image, with colors denoting the tumor, immune, myeloid, stromal, and fibroblast classes listed in the legend. (**E**) Enlarged lymphoid-myeloid neighborhood highlighting mregDCs, T cells, macrophages, CAFs, and inflammatory tumor cells. (**F**) Second enlarged neighborhood illustrating the same TLS-associated mregDC/AP spatial architecture.

**Figure 5:**
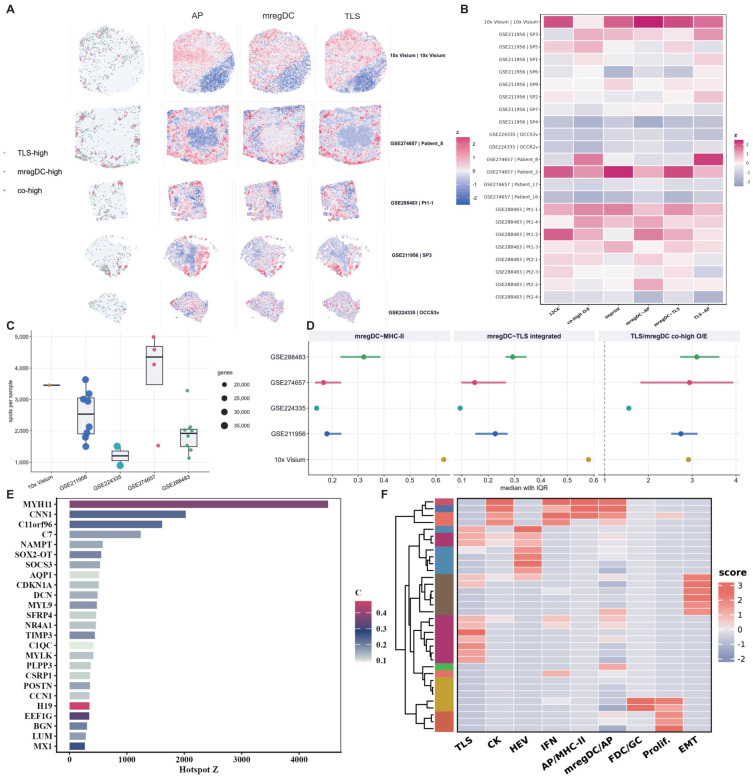
Multi-sample spatial transcriptomics supports recurrent mregDC/TLS and AP-proxy program coupling. (**A**) Representative public ovarian spatial transcriptomic maps showing AP/MHCII-proxy, mregDC, and TLS score distributions together with TLS-high, mregDC-high, and co-high spots across selected samples; green, purple, and pink points denote TLS-high, mregDC-high, and co-high spots, respectively, whereas score-map gradients are defined by the adjacent color scale. (**B**) Sample-level standardized heatmap of TLS 12-CK, TLS imprint, mregDC–AP/MHC-II, mregDC–TLS, TLS–AP/MHC-II, and co-high observed/expected metrics. (**C**) Multi-sample spatial cohort summary showing spots per sample and gene-panel size. (**D**) Dataset-level spatial support estimates for mregDC–MHC-II, mregDC–TLS, and TLS/mregDC co-high enrichment. (**E**) Hotspot-ranked spatially autocorrelated genes in sampled spatial data. (**F**) TLS-field module heatmap summarizing TLS, chemokine, HEV/stromal, IFN, MHC-II/AP, mregDC, FDC/GC, proliferation, and EMT/invasion programs; red and blue tiles denote higher and lower standardized values, respectively, and neutral tiles denote zero-centered or invariant readouts rather than missing data.

**Figure 6:**
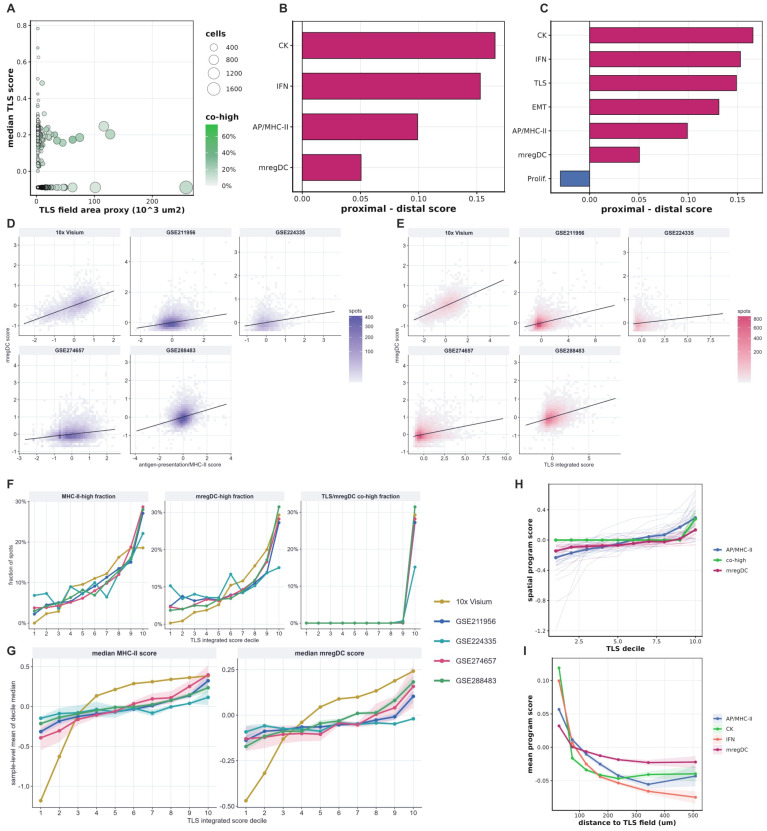
TLS-decile and distance-gradient analyses summarize spatial co-variation. (**A**) Relationship between TLS field-area proxy and median TLS score, with co-high fraction and cell count encoded by color and size. (**B**) Proximal-to-distal module contrast for cell-state-associated programs near TLS fields. (**C**) Proximal-to-distal module contrast for immune, antigen-presentation, IFN, and tumor-program scores; magenta bars indicate positive proximal-minus-distal contrasts and blue bars indicate negative contrasts. (**D**) Spot-level scatter matrices linking TLS-integrated and mregDC-associated scores across selected spatial samples. (**E**) Spot-level scatter matrices linking TLS-integrated and AP/MHC-II-proxy scores. In (**D**,**E**), point intensity reflects local spot density and black lines are linear fits. (**F**) TLS-decile gradients for MHC-II-high, mregDC-high, and TLS/mregDC co-high fractions; line colors denote the five public datasets listed in the legend. (**G**) Dataset-level TLS-decile gradients for median MHC-II and mregDC scores using the same dataset colors. (**H**) TLS-decile program trends in the Xenium section; colored lines denote AP/MHC-II, co-high, and mregDC programs, and pale lines show individual field-level trajectories. (**I**) Mean program-score gradients as a function of distance from TLS-score-defined fields; colored lines denote AP/MHC-II, cytokeratin/tumor (CK), IFN, and mregDC programs, and shaded bands show the corresponding uncertainty summaries.

To provide representative tissue-image support for the transcript-defined mregDC state, multiplex immunofluorescence was refocused on CD11C, HLA-DRA, LAMP3, and CCR7 (Figure 3). CD11C and HLA-DRA mark APC-enriched regions, whereas LAMP3 and CCR7 identify the mature migratory mregDC program in the context of the transcriptomic evidence. This four-marker panel was used to visualize mregDC-associated staining within tissue regions; TLS identity was defined separately by the 12-chemokine score, TLS-imprint modules, and spatial co-localization analyses in Figure 4, Figure 5 and Figure 6, with gene lists and coverage audits provided in Supplementary Table S2. Image-based review of representative merged IF fields included one case per cancer type and one selected ROI per case (OVC, CEAD, CESC, and UCEC; 4 ROIs total). Raw ROI counts identified 19, 9, 15, and 65 CD11C/HLA-DRA-positive APC-like events in the OVC, CEAD, CESC, and UCEC ROIs, respectively, with 15, 3, 10, and 53 merged-image ROI events scored as regional four-channel co-occurrence. Figure 3E displays the corresponding selected-crop image-derived event densities, calculated from these raw counts and the displayed crop areas. Figure 3F displays the regional four-channel events as a fraction of APC-like events (78.9%, 33.3%, 66.7%, and 81.5%, respectively). Because this assessment used flattened merged images rather than raw separated channels, these values are descriptive selected-crop metrics and should not be interpreted as case-level tissue density, cell-resolved four-marker positivity, formal co-localization statistics, or cross-cancer abundance differences. Additional scRNA-seq and IF support panels summarizing sample composition, marker weights, marker-score source summaries, and IF raw counts are provided in Supplementary Figure S8.

### 3.4. Xenium Segmentation Localizes mregDC/AP Signals to TLS-Score-Defined Spatial Fields

Xenium spatial transcriptomics resolved 407,124 cells in a public OVC tissue section and enabled spatial analysis of TLS, mregDC, and antigen-presentation programs (Figure 4). TLS-score maps, mregDC-associated maps, and co-localization contours identified immune-enriched fields with overlapping TLS and mregDC/AP signals. Cell-class segmentation further showed that these fields were embedded within a mixed tumor-immune-stromal architecture rather than distributed randomly across the section. Additional Xenium spatial maps for TLS, mregDC, AP/MHC proxy, IF-core proxy, non-random genes, and TLS/mregDC co-high cells are provided in Supplementary Figures S3 and S9, with TLS-field object and gene-coverage audits shown in Supplementary Figure S10.

Neighborhood visualizations showed mregDC/AP signals positioned within or adjacent to lymphoid and myeloid neighborhoods in this single public OVC Xenium section, illustrating proximity of mregDC-associated and antigen-presentation-proxy signals to TLS-score-defined regions. Because the Xenium panel did not contain exact HLA-DRA/HLA genes, public spatial analyses used antigen-presentation/MHC-II proxy genes when available, while direct HLA-DRA evidence was assigned to the IF layer. Thus, Figure 4 establishes spatial localization and neighborhood organization in one exemplar section, not histological TLS maturation staging, direct HLA-DRA transcript measurement, or cross-sample recurrence.

### 3.5. Multi-Sample Spatial Transcriptomics Supports Recurrent mregDC/TLS and Antigen-Presentation Program Coupling

We next tested whether the spatial coupling observed in the Xenium section recurred across additional ovarian spatial transcriptomic samples (Figure 5). Across 23 public full-transcriptome spatial samples comprising 56,546 spots, mregDC, TLS, and antigen-presentation/MHC-II proxy programs were scored per spot using available genes, including exact HLA-DRA, LAMP3, and CCR7 where present. The broad spatial TLS-imprint score used an expression-overlap imprint under a harmonized label rather than assuming complete recovery of the patient-level 29-gene imprint in every platform (Supplementary Table S2). Selected spatial maps showed recurrent co-occurrence of TLS-high, mregDC-high, antigen-presentation-high, and TLS/mregDC co-high regions across independent datasets. Because gene coverage varied by platform, these analyses support program-level consistency rather than direct validation of the exact four-marker IF phenotype in every dataset.

Sample-level summaries showed positive median coupling between mregDC and TLS programs and recurrent enrichment of TLS/mregDC co-high spots above random expectation. Hotspot and module-support analyses further showed spatially structured stromal, inflammatory, and lymphoid-associated features in the same spatial framework. Dataset-level spatial-support and TLS-gradient summaries are provided in Supplementary Figures S4 and S11. These multi-sample spatial analyses support recurrent program-level coupling among TLS, mregDC, and antigen-presentation modules, while remaining transcriptomic support analyses rather than histological TLS-staging assays.

### 3.6. TLS-Proximal Fields Show Distance-Dependent Enrichment of mregDC, MHC-II/AP, and Interferon Programs

To move beyond simple co-localization, we analyzed the spatial relationship between TLS-score-defined fields and mregDC/AP programs using decile and distance-gradient summaries (Figure 6). TLS-high regions were enriched for mregDC-high and antigen-presentation/MHC-II proxy-high signals, and proximal-to-distal analyses showed higher mregDC, MHC-II/AP proxy, and interferon-associated programs near TLS fields. Spot-level scatter matrices also supported coordinated variation among TLS-integrated, mregDC, and antigen-presentation scores.

Across multi-sample spatial datasets, TLS-ranked deciles showed increasing mregDC-high and antigen-presentation-high fractions in higher TLS deciles, whereas distance-to-TLS-field curves showed decreasing co-high or immune-program support away from TLS regions. These results refine the main spatial conclusion from “TLS and mregDC co-localize” to a structured field model in which TLS-positive regions contain or border antigen-presentation-enriched mregDC signals. Because distance-gradient summaries were not designed as causal or fully immune-richness-adjusted tests, they describe spatial co-variation; specificity was therefore evaluated separately with immune-richness-adjusted analyses.

### 3.7. Ten-Method Immune Deconvolution Links the TLS/mregDC Axis to an Immune-Infiltrated Patient Contexture

**Figure 7:**
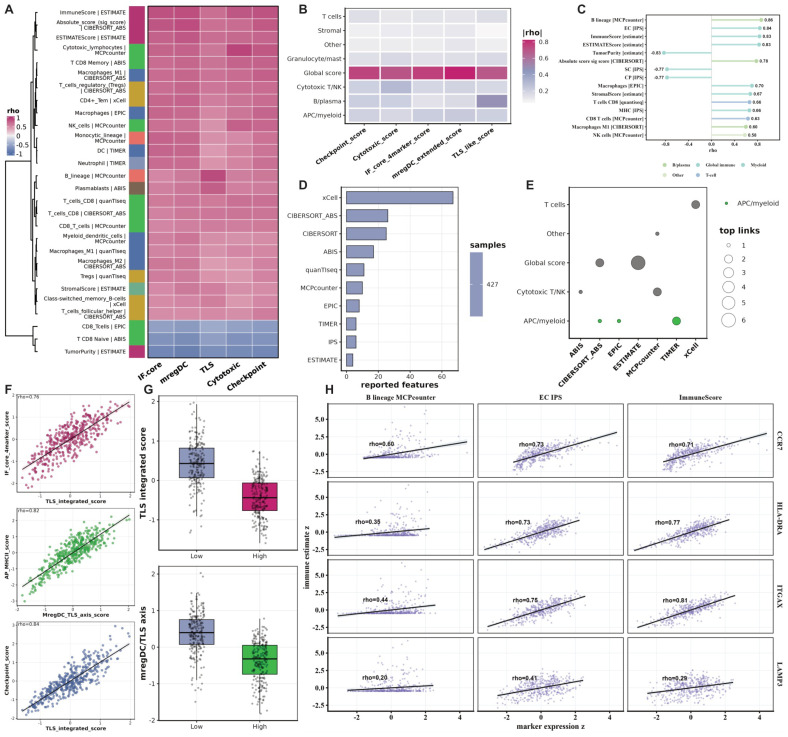
Ten-method immune deconvolution links the TLS/mregDC program to immune infiltration in TCGA-OV. (**A**) Hierarchical heatmap of immune-estimate correlations with IF-core, mregDC, TLS, and checkpoint scores across deconvolution methods. (**B**) Immune-class summary heatmap comparing lymphoid, myeloid, stromal, cytotoxic, and APC-related classes. (**C**) Ranked immune estimates associated with the TLS/mregDC axis; dot color denotes immune class, horizontal position denotes the Spearman correlation, and intervals show the corresponding uncertainty summary. (**D**) Method coverage and number of reported immune features. (**E**) Method-class coverage map across broad immune compartments. (**F**) Correlation scatter panels for TLS-integrated, mregDC–TLS-axis, and checkpoint-associated scores; points are TCGA-OV patients and lines are fitted trends. (**G**) Distribution of TLS/mregDC-associated scores by low and high patient groups; boxes show the median and interquartile range and points represent patients. (**H**) Marker-expression scatter matrices linking immune-cell estimates with representative mregDC/AP marker expression; pale-purple points represent patient-level values, black lines show fitted trends, and the annotated *ρ* values are Spearman correlations.

We next evaluated whether the spatially localized TLS/mregDC program was reflected in bulk immune contexture. In TCGA-OV, tumors were analyzed with the ten-method deconvolution framework comprising quanTIseq, TIMER, CIBERSORT, CIBERSORT-ABS, MCP-counter, xCell, EPIC, ESTIMATE, ABIS, and ConsensusTME (Figure 7). Because TLS scores intrinsically contain B-cell and lymphoid-chemokine information, we prioritized immune-richness-adjusted analyses over the largest raw correlations. After adjustment for broad immune context, tumor purity, B-cell, and T-cell controls, mregDC–TLS and mregDC–AP/MHC-II associations remained positive but modest in TCGA-OV (partial ρ=0.288, $p=1.4\times 10^{-9}$ and partial ρ=0.249, $p=1.8\times 10^{-7}$, respectively; n=427). Unadjusted ten-method correlations were higher, as expected for immune-rich tumors; for example, MCP-counter B-lineage estimates showed the strongest raw association with the TLS-integrated score (ρ=0.86), and ESTIMATE immune scores, TIMER dendritic-cell estimates, ABIS plasmablast estimates, and multiple cytotoxic/myeloid estimates were also positively associated, whereas tumor purity was negatively associated.

Program-level immune-class summaries showed consistent positive correlations across B/plasma, T-cell, myeloid, global-immune, and DC/APC classes. Spearman correlations across the ten-method deconvolution layer were corrected using the Benjamini–Hochberg procedure across 860 non-missing feature–target tests; adjusted values are provided in the source data. The method-coverage summary showed that deconvolution evidence was not driven by a single algorithm, while marker-expression scatter panels linked CCR7, HLA-DRA, ITGAX, and LAMP3 to immune-estimate variation. In multi-sample spatial spots, mregDC–TLS and mregDC–AP/MHC-II relationships also remained positive after local B/T/AP or B/T/TLS controls (partial ρ=0.135 and 0.136, both $p<10^{-4}$; n=41,867). In the Xenium cell-level sensitivity analysis, strict adjustment primarily retained mregDC–AP/MHC-II coupling (partial ρ=0.153, $p<10^{-4}$), whereas the mregDC–TLS residual correlation was very small in magnitude (partial ρ=−0.025 despite a nominal $p<10^{-4}$ due to large cell number). Additional specificity-adjusted association checks and ten-method deconvolution summaries are shown in Supplementary Figures S6 and S12. Taken together, these association tests indicate a measurable mregDC/AP component within the TLS-associated program, while cautioning against over-interpreting cell-level TLS coupling as independent of local antigen-presentation, interferon, and tumor-program gradients.

### 3.8. Patient-Level Composite Scores Connect the TLS/mregDC Axis to Exploratory Outcome Stratification

**Figure 8:**
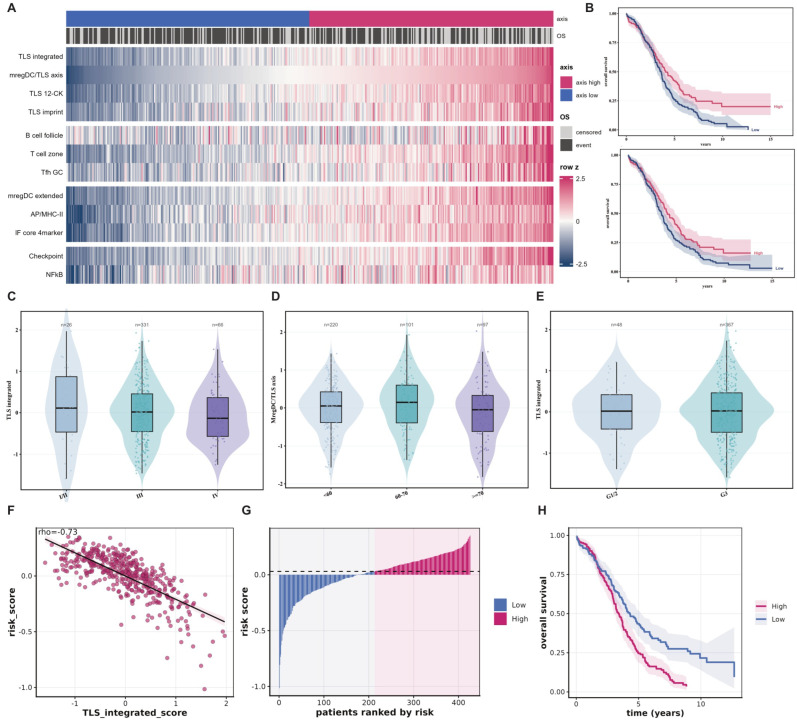
Patient-level composite scoring links the TLS/mregDC axis to exploratory outcome stratification. (**A**) Patient-level feature matrix integrating TLS, mregDC, antigen-presentation/MHC-II, B-cell, T-cell, checkpoint, IF-core, and NF-κB-associated programs; heatmap colors denote standardized feature values. (**B**) Kaplan–Meier curves comparing patient groups defined by TLS/mregDC-axis features; line colors identify the groups shown in each panel and shaded bands are 95% confidence intervals. (**C**) TLS-integrated-score distributions across the plotted T-stage categories. (**D**) mregDC/TLS-axis-score distributions across the plotted age groups. (**E**) TLS-integrated-score distributions across the plotted grade groups. In (**C**–**E**), points represent patients and boxes show the median and interquartile range. (**F**) Relationship between TLS-integrated score and derived risk score; points represent patients and the line is the fitted trend. (**G**) Risk-score waterfall with the vertical cutoff, high/low grouping backgrounds, and ordered patients. (**H**) Kaplan–Meier analysis of internally defined risk groups in TCGA-OV (High: n=213, events = 155; Low: n=213, events = 110; HR = 1.62, 95% CI 1.27–2.07; Cox $p=1.24\times 10^{-4}$; log-rank $p=1.08\times 10^{-4}$). These patient-level panels are exploratory and internally assessed.

To translate cell-state and spatial findings into patient-level features, we integrated IF-core, mregDC, antigen-presentation/MHC-II proxy, TLS-module, B-cell, T-cell, Tfh/GC, checkpoint, and NF-κB scores into a patient-level composite framework (Figure 8). The objective of this analysis was to test whether the spatial TLS/mregDC/AP program could be summarized at the patient level in TCGA-OV, not to define a clinically validated diagnostic or prognostic assay. TLS-integrated and mregDC/TLS-axis features were strongly co-structured with B-cell follicle, T-cell zone, checkpoint, and antigen-presentation/MHC-II proxy programs. In the current TCGA-OV analysis, higher TLS/mregDC composite support was associated with lower derived risk, whereas the internally defined high-risk group showed poorer survival. In the internally defined risk-group comparison, high-risk patients (n=213; events = 155) had poorer overall survival than low-risk patients (n=213; events = 110; HR = 1.62, 95% CI 1.27–2.07; Cox $p=1.24\times 10^{-4}$; log-rank $p=1.08\times 10^{-4}$).

Clinical-stratum analyses did not support a robust FIGO- or grade-dependent TLS-score shift, although age-associated variation was observed for selected TLS-imprint and antigen-presentation/MHC-II proxy scores. These results position the TLS-associated mregDC/AP field as a patient-level immune context rather than a stand-alone clinical biomarker.

### 3.9. Exploratory LASSO-Cox and Model-Validation Analyses Summarize a TLS/mregDC Immune-Prognostic Feature Set

**Figure 9:**
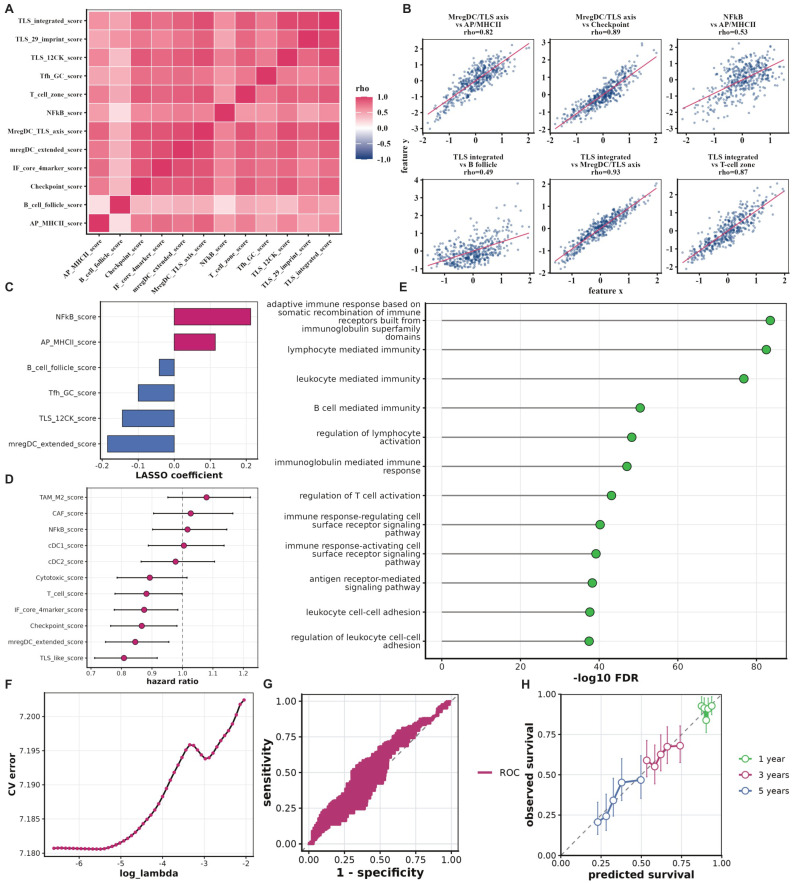
Exploratory LASSO-Cox modeling prioritizes a TLS/mregDC immune-prognostic feature set. (**A**) Correlation heatmap of patient-level TLS, mregDC, antigen-presentation/MHC-II, IFN, checkpoint, and NF-κB-related features; red and blue denote positive and negative correlations, respectively. (**B**) Scatter matrix of key composite-score relationships; blue points represent patients and red lines are fitted linear trends. (**C**) LASSO-Cox selected feature coefficients; bar colors distinguish positive from negative coefficients. (**D**) Feature-level Cox forest plot for the plotted immune, dendritic-cell, stromal, and TLS/mregDC-associated scores; points denote hazard ratios, horizontal intervals denote 95% confidence intervals, and the dashed vertical line marks a hazard ratio of 1. (**E**) Pathway enrichment of model-associated immune programs; horizontal position denotes −log_10_ false-discovery rate for each pathway. (**F**) LASSO-Cox cross-validation-error trajectory across the plotted log-lambda values; magenta points and the black connecting line show the mean cross-validation error. (**G**) Overlaid 1-, 3-, and 5-year time-dependent receiver-operating-characteristic traces for exploratory internal model assessment, displayed using the shared magenta rendering in the panel. (**H**) Calibration summaries by risk-score quintile at 1, 3, and 5 years.

We then evaluated whether the patient-level TLS/mregDC framework could be summarized by an exploratory immune-prognostic model (Figure 9). Correlation and scatter-matrix analyses showed coordinated variation among TLS-integrated, mregDC/TLS-axis, MHC-II/AP, IFN, checkpoint, and NF-κB-related features. LASSO-Cox feature selection retained mregDC, antigen-presentation/MHC-II, TLS-12CK, B-cell follicle, Tfh/GC, and NF-κB scores. In a GDC clinical sensitivity analysis, the risk score remained associated with overall survival after adjustment for age, advanced FIGO stage (III/IV vs. I/II), and high grade (G3/G4/GB vs. G1/G2) (HR = 4.62, 95% CI 2.30–9.25, $p=1.6\times 10^{-5}$; n=416). Age was also independently associated with outcome in this model, whereas advanced stage and high grade were not independently significant, consistent with the limited stage/grade variance in TCGA-OV and indicating that this sensitivity analysis tests whether the risk-score association is abolished by available covariates rather than establishing independence from the full clinical picture. Residual disease was not included in the adjusted model because it was recorded for only 14 of 426 model patients.

Pathway analysis of the high-risk-associated expression program highlighted immune response, regulation of T-cell activation, leukocyte migration, and inflammatory pathways. Time-dependent ROC, LASSO-Cox cross-validation, calibration-by-risk-quintile analyses, and the GDC clinical sensitivity model were retained as internal model-performance checks. Additional model-validation and patient-level composite panels, including time-dependent ROC, LASSO-Cox cross-validation, calibration, decision-curve summaries, risk-score distributions, and feature-correlation structure, are shown in Supplementary Figures S5 and S13. Because this model was trained and assessed within TCGA-OV without an independent prospective cohort, it is reported as exploratory prognostic stratification rather than a clinically deployable predictor.

### 3.10. scTenifoldKnk and CellOracle Prioritize Regulatory Nodes of the TLS/mregDC Antigen-Presentation Program

**Figure 10:**
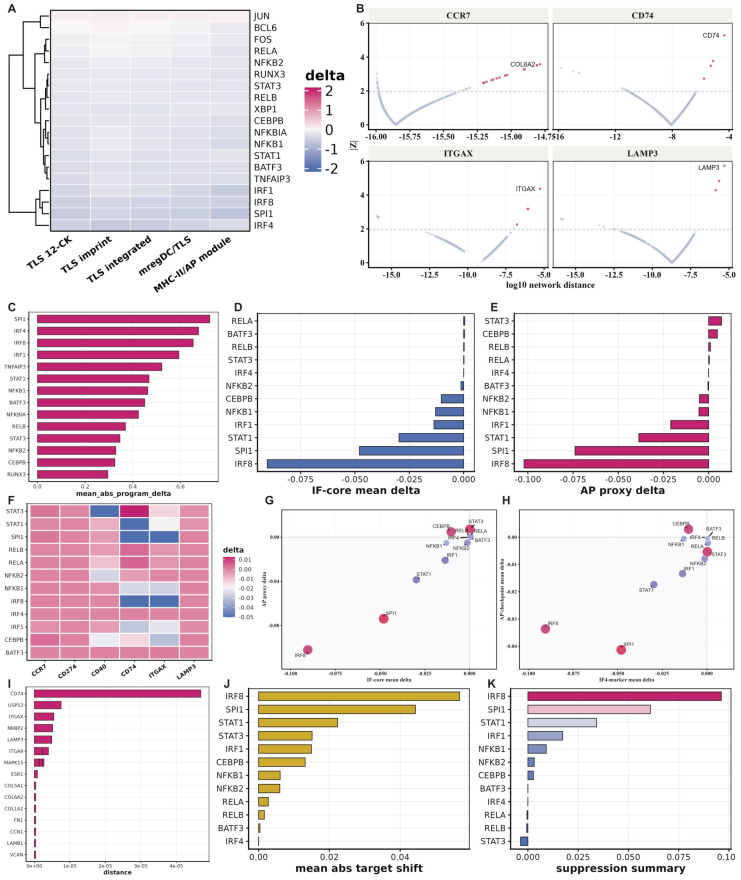
scTenifoldKnk and CellOracle prioritize candidate regulatory nodes of the TLS/mregDC program. (**A**) CellOracle TF-level perturbation heatmap across the TLS 12-CK, TLS imprint, TLSintegrated, mregDC/TLS, and MHC-II/AP modules; red and blue denote positive and negative program deltas. (**B**) scTenifoldKnk virtual-knockout distance curves for CCR7, CD74, ITGAX, and LAMP3; pale-blue points and curves show background network-distance behavior, magenta points highlight the most shifted targets, and dashed horizontal lines mark the plotted reference threshold. (**C**) Perturbation-burden ranking based on mean absolute program delta. (**D**) IF-core mean-delta summary for prioritized regulators; blue bars denote negative deltas. (**E**) AP-proxy mean-delta summary; magenta bars denote the signed deltas. (**F**) TF-by-target perturbation matrix for CCR7, CD274, CD40, CD74, ITGAX, and LAMP3. (**G**) TF-level scatter plot relating IF-core mean delta to AP-proxy delta. (**H**) Corresponding four-marker summary relating IF four-marker mean delta to AP four-marker mean delta. In (**G**,**H**), labels identify prioritized TFs and point color and size encode the plotted shift magnitude. (**I**) KO-sensitive target ranking from marker/proxy virtual perturbation, with network distance shown in explicit scientific notation. (**J**) Mean absolute target-shift summary. (**K**) Target-suppression summary for prioritized regulatory nodes.

We then used scTenifoldKnk and CellOracle analyses to prioritize candidate regulators of the mregDC program (Figure 10). scTenifoldKnk virtual knockout was completed for ITGAX, LAMP3, CCR7, and CD74 using the public 10x scFFPE matrix. CD74 was retained as the antigen-presentation/MHC-II proxy because exact HLA-DRA/HLA genes were absent from the matrix. CCR7 virtual knockout produced a prominent network-shift signal, and KO-sensitive target summaries highlighted marker and regulatory genes affected by the tested perturbations.

CellOracle 0.20.0 prioritized TF-level perturbations among NF-κB/antigen-presentation-associated regulators. IRF8, SPI1, STAT1, IRF1, and NFKB1 showed prominent shifts in IF-core or antigen-presentation proxy summaries, and cross-method integration ranked IRF8 and SPI1 among high-priority regulators across CellOracle and patient-level virtual-KO estimates. Marker-level virtual knockout was therefore handled by scTenifoldKnk, whereas CellOracle estimated upstream transcription-factor perturbation effects on downstream marker and antigen-presentation/MHC-II response modules. These computational results nominate experimentally testable regulators and should not be interpreted as direct mechanistic proof.

### 3.11. Exploratory Drug-Prioritization Analyses Are Provided as Supplementary Target-Class Hypotheses

Because drug screening is peripheral to the central TLS/mregDC spatial question, the drug-prioritization layer was moved to the Supplementary Materials as an exploratory prioritization output downstream of the perturbation analyses (Supplementary Figure S7). Integrated TLS/mregDC perturbation scores prioritized IRF8 and SPI1, whereas RELA/RELB/NFKB2/NFKB1 mapped to IKK/NF-κB-related classes and STAT1/STAT3 mapped to JAK/STAT-related classes. The virtual-screening rank favored JAK-inhibitor candidates for predicted high-risk signature reversal, while proteasome-related candidates had lower predicted reversal scores in the current model. Most ranked compounds are not approved ovarian-cancer therapies, and this analysis did not test whether PIK3CA mutation status explained cell-line sensitivity to any PI3K-pathway inhibitor, including inavolisib, because the deposited drug-prioritization source data contain target/program-level prioritization features rather than matched pharmacogenomic IC50 and PIK3CA-annotation tables.

Vina redocking completed three non-covalent receptor-ligand pairs: ruxolitinib–JAK2, baricitinib–JAK2, and carfilzomib–PSMB5. Bortezomib and ixazomib pairs were marked Vina-ineligible under the current non-covalent Vina setup because boronate chemistry is not adequately handled by standard atom typing. These results are not part of the primary evidence chain for the TLS/mregDC spatial model and should be used only to prioritize compounds or target classes for experimental testing, not to infer drug efficacy, ovarian-cancer clinical relevance, mutation-defined response, or regulatory-phenotype reversal.

## 4. Discussion

This study reframes the OVC mregDC finding as a spatial association between an operational TLS program and a $LAMP3^{+}CCR7^{+}$ mature migratory DC state rather than as a single-marker observation. Across single-cell analysis, representative four-marker immunofluorescence, Xenium segmentation, multi-sample spatial support, TLS-distance gradients, immune deconvolution, patient-level modeling, in silico perturbation, and drug prioritization, the $LAMP3^{+}CCR7^{+}$ dendritic-cell state showed features consistent with mature migratory mregDCs and localized to operational TLS-score-defined regions. The most important conceptual shift is that the spatial evidence is not limited to co-localization: TLS-field segmentation and distance-gradient analyses support a model in which TLS-positive regions contain or border mregDC/AP-enriched fields with antigen-presentation/MHC-II proxy and interferon-associated programs. Immune-richness-adjusted analyses further support a non-redundant mregDC/AP component at patient and spatial-spot levels. At a single-cell resolution, however, residual mregDC–TLS coupling in the Xenium section was negligible after adjustment (partial ρ=−0.025), indicating that the cell-level TLS association is largely attributable to local immune-program richness; the field model should therefore be read as a program-level spatial association rather than as a proven discrete cellular niche.

The single-cell results are consistent with prior reports that canonical cDC1 and cDC2 populations can acquire $LAMP3^{+}$ regulatory/migratory DC programs in tumors [11,13,40,46]. However, the trajectory panels remain state-relationship analyses and should not be interpreted as definitive lineage conversion or a causal maturation sequence. Similarly, the IF panel provides representative tissue-image support for regional co-occurrence of CD11C, HLA-DRA, LAMP3, and CCR7 staining consistent with mregDC-associated immune-region staining across gynecological tumor types, but single-cell four-marker co-positivity was not quantified, and TLS identity itself was assigned from transcriptomic and spatial TLS programs. This separation is important because histological TLS maturation would ideally require additional B-cell follicle, T-cell zone, FDC/germinal-center, and HEV markers beyond the four-marker mregDC IF panel.

The revised interpretation is aligned with recent TLS literature. The pan-cancer spatial TLS atlas in Science showed that TLS biology is better captured by maturation state, spatial location, distance-dependent tumor/immune gradients, and maturation-aware composite metrics than by binary TLS presence alone [17]. The same atlas provides an important boundary for OVC interpretation: TLSs were limited in the OVCA spatial-transcriptomic subset, whereas the HGSOC H&E therapy cohort contained many segmented TLSs and showed directionally favorable associations between mature TLS composition and treatment response [17]. Our spatial figures therefore treat TLS-field segmentation, TLS/mregDC/AP co-localization, multi-sample spatial support, and TLS-distance gradients as the core spatial evidence in TLS-positive OVC regions rather than as evidence that TLSs are uniformly abundant across OVC. The ovarian-cancer TLS review and integrated tumor-associated TLS spatial profiling further support using B-cell, T-cell, FDC/germinal-center, HEV/stromal, chemokine, interferon, and antigen-presentation modules rather than relying on a single marker or chemokine score [18,19]. A 2025 mature-TLS study linked mature TLSs with intratumoral T- and B-cell responses, consistent with a productive immune niche model, whereas spatial tryptophan-metabolism analysis cautions that TLS-rich fields can remain functionally constrained when local metabolic programs restrict TLS maturation [61,62]. Accordingly, the claims here are deliberately framed around a TLS-associated mregDC/AP-proxy program and its immune-prognostic context, not around definitive histological TLS staging or therapeutic efficacy.

This framework also clarifies the biological relationship between mregDCs and TLSs. TLSs are multicellular tissue structures requiring organized B-cell, T-cell, FDC/germinal-center, stromal/HEV, chemokine, and antigen-presentation components, whereas mregDCs represent a mature migratory APC state that may participate in, border, or reflect these immune fields. Therefore, a CD11C/HLA-DRA/LAMP3/CCR7 mregDC/AP panel cannot replace histological TLS assessment. It could, however, become a simpler pathology triage layer: mregDC-positive APC fields may identify sections that should undergo full TLS staging, additional B/T/FDC/GC/HEV staining, or spatial profiling. This distinction is clinically important because mregDC programs can carry both antigen-presentation and regulatory features, including checkpoint and Treg-associated axes, and should not be interpreted as uniformly beneficial without context [63,64,65,66].

The translational implication is that the mregDC/TLS axis may help select, monitor, and rationally combine immunotherapies rather than serving as a stand-alone treatment target at this stage. DC vaccines have already been explored in ovarian cancer, but durable benefit has been limited by antigen selection, DC maturation state, immune suppression, and lack of robust tissue biomarkers [67]. A TLS-associated mregDC/AP readout could help identify patients with pre-existing local antigen-presentation niches, monitor whether vaccination or chemo-antiangiogenic treatment expands mature APC/TLS programs, and guide combinations with checkpoint blockade, anti-VEGF therapy, or stromal/metabolic remodeling. Engineered DC or CAR-DC strategies are conceptually attractive because they could combine tumor targeting, antigen presentation, cytokine delivery, and T-cell priming, and recent engineered-myeloid and CAR-DC work supports this direction preclinically [68,69]. For OVC, however, this remains a future experimental path: the current data nominate mregDC/TLS-associated biology as a biomarker and perturbation-prioritization framework, not as evidence that DC vaccines or CAR-DC products are clinically ready for this indication.

The patient-level analyses connect the spatial program to a broader immune contexture. Ten-method deconvolution indicated that TLS/mregDC-score-high tumors were associated with B/plasma, myeloid/APC, T-cell, cytotoxic/NK, and global immune-infiltration programs, while the composite patient-level score and LASSO-Cox analyses provided exploratory outcome stratification. These findings are compatible with the coexistence of organized immune infiltration and immune-regulatory restraint in OVC [68,70,71,72,73,74,75,76,77]. The adjusted analyses reduce, but do not eliminate, the concern that the TLS/mregDC/AP signal reflects global immune infiltration. The strongest adjusted support was observed for mregDC–TLS and mregDC–AP/MHC-II coupling at the TCGA and multi-sample spot levels, whereas the Xenium cell-level analysis mainly preserved mregDC–AP/MHC-II coupling. Therefore, the field model is best interpreted as a spatially organized immune-program association that requires additional raw-channel IF, histological TLS staging, and functional perturbation for definitive validation. The exploratory risk score remained associated with outcome in an age/stage/grade sensitivity Cox model, although stage and grade were not independently significant in this TCGA-OV sensitivity cohort and residual disease was too sparsely recorded in the GDC clinical fields for adjustment. Because the prognostic model was derived and internally assessed in TCGA-OV, it should be treated as an exploratory immune-prognostic feature set rather than as a deployable clinical test.

The perturbation and drug-prioritization layers should be read as hypothesis prioritization. scTenifoldKnk tested marker/proxy network disruption after virtual knockout of ITGAX, LAMP3, CCR7, and CD74, whereas CellOracle prioritized upstream transcription factors, including IRF8, SPI1, STAT1, IRF1, and NFKB1, in a TF-level perturbation framework. The supplementary drug-prioritization layer (Supplementary Figure S7) then connects these regulators to targetable classes and docking feasibility, but it does not provide pharmacological validation, in vitro blockade, actual genetic knockout, co-culture evidence, or cell-line drug-response testing. In particular, docking scores and virtual-screening ranks nominate compounds and target classes for future testing; they do not establish efficacy, mechanism, mutation-defined sensitivity, regulatory-phenotype reversal, or clinical utility. Many ranked compounds are approved or studied mainly outside ovarian cancer, including autoimmune, hematologic, or experimental contexts, so this layer should be treated as a target-class hypothesis generator rather than as an ovarian-cancer therapeutic recommendation.

Several limitations frame the interpretation of this study. First, this study relies substantially on public single-cell, spatial-transcriptomic, and bulk-transcriptomic datasets. These resources are valuable for integration and hypothesis generation, but they are static datasets and cannot establish temporal tumor progression, dynamic TLS maturation, treatment-induced remodeling, or causality. Second, the representative IF layer included one case and one ROI per cancer type and was reviewed on merged flattened images. The selected-crop event densities and regional co-occurrence fractions shown in Figure 3 are descriptive image-derived metrics; they do not support a cross-cancer statistical comparison, case-level whole-tissue density estimate, or raw-channel single-cell co-localization assay. The scRNA-seq analysis identifies a small transcript-defined $LAMP3^{+}CCR7^{+}$ DC state, whereas the IF fields were selected immune-enriched representative regions and should not be interpreted as whole-tissue mregDC abundance. Third, the public Xenium and scFFPE transcriptomic matrices did not contain exact HLA-DRA/HLA-family genes, so public-data analyses support antigen-presentation/MHC-II proxy signals rather than direct HLA-DRA expression. TLS assignment was operational and based on a 12-chemokine module, a patient-level 29-gene TLS imprint, expression-overlap spatial TLS-imprint support, and spatial co-localization rather than full histological staging of TLS maturation. Immune-richness-adjusted partial correlations were added as confounding audits, but they cannot substitute for prospective multiplex tissue cohorts, raw-channel co-localization statistics, or perturbation experiments. The bulk and survival analyses were retrospective, and the computational perturbation and drug-prioritization analyses require functional validation. Overall, the manuscript provides a multimodal, hypothesis-generating framework for future functional, spatial, and translational studies of the mregDC/TLS axis in OVC.

## 5. Conclusions

Overall, our multimodal analyses support a spatial association between a $LAMP3^{+}CCR7^{+}$ mregDC program and operational TLS-score-defined regions with antigen-presentation/MHC-II proxy support in TLS-positive OVC regions. The integrated single-cell, representative IF, Xenium, multi-sample spatial, distance-gradient, immune-deconvolution, immune-richness-adjusted, patient-level modeling, scTenifoldKnk, and CellOracle data are consistent with a spatially organized mature migratory DC program and nominate NF-κB/antigen-presentation-associated regulatory, biomarker, vaccine-monitoring, and experimental perturbation axes for future validation. The virtual-screening and docking analyses are retained only as exploratory target-class prioritization and do not establish ovarian-cancer therapeutic efficacy.

## Data Availability

The public scRNA-seq datasets analyzed in this study are available from GEO under accession numbers GSE197461, GSE208653, and GSE173682. The multi-sample spatial analysis used one 10x Visium ovarian FFPE dataset and GEO spatial datasets GSE211956, GSE224335, GSE274657, and GSE288483, with per-sample identifiers listed in Supplementary Table S3. The public 10x Genomics scFFPE ovarian cancer matrix and Xenium Prime FFPE human ovarian cancer dataset are available from 10x Genomics. TCGA-OV RNA-seq and clinical data were retrieved from the Genomic Data Commons, and normal-tissue reference data were retrieved from GTEx. Derived source-data tables for Figure 1, Figure 2, Figure 3, Figure 4, Figure 5, Figure 6, Figure 7, Figure 8, Figure 9 and Figure 10 and Supplementary Figures S1–S14 are included in the accompanying Source Data archive, where table output was generated and mapped in SOURCE_DATA_INDEX.csv. Analysis scripts, figure scripts, source-data mapping files, and code materials are archived at Zenodo (https://doi.org/10.5281/zenodo.21184531) and available at GitHub (https://github.com/Luciky-Leo/Luciky-Leo-mregdc-tls-ov, accessed on 4 July 2026).

## Supplementary Materials

The following supporting information can be downloaded at: https://www.mdpi.com/article/10.3390/cancers18142259/s1. Figure S1: singleCellHaystack spatial non-randomness audit; Figure S2: Hotspot spatial-autocorrelation audit; Figure S3: representative spatial core of the TLS/mregDC/AP-proxy niche; Figure S4: multi-sample spatial validation and gene-coverage audit; Figure S5: internal patient-level model validation; Figure S6: immune-context-adjusted specificity analysis; Figure S7: exploratory drug-prioritization layer; Figure S8: scRNA-seq and representative IF support; Figure S9: Xenium program maps in a common spatial coordinate system; Figure S10: TLS-field segmentation and program coverage; Figure S11: TLS distance and decile gradients; Figure S12: ten-method immune-deconvolution context; Figure S13: patient-level composite feature structure; Figure S14: virtual perturbation support. Table S1: scRNA-seq GEO sources; Table S2: signature definitions and gene coverage; Table S3: spatial and 10x dataset accessions. Source Data for the main and supplementary figures are provided as a separate archive and in the public repository. Figures S7 and S14 provide exploratory computational drug-prioritization, docking, and virtual-perturbation hypotheses and are not primary evidence for the TLS/mregDC spatial conclusion.

## Abbreviations

The following abbreviations are used in this manuscript:

APCs	antigen-presenting cells
AUC	area under the curve
CAFs	cancer-associated fibroblasts
CCR7	C-C motif chemokine receptor 7
CEAD	cervical adenocarcinoma
CESC	cervical squamous cell carcinoma
CTLA4	cytotoxic T lymphocyte associated protein 4
DCs	dendritic cells
FFPE	formalin-fixed paraffin-embedded
GEO	Gene Expression Omnibus
GRN	gene-regulatory network
H&E	hematoxylin and eosin staining
HR	hazard ratio
IF	immunofluorescence
IKK	inhibitor of nuclear factor kappa-B kinase
JAK	Janus kinase
ML	machine learning
mregDCs	mature dendritic cells enriched in immunoregulatory molecules
NF-κB	nuclear factor kappa-light-chain-enhancer of activated B cells
OVC	ovarian cancer
PDB	Protein Data Bank
PD-L1	programmed cell death ligand 1
PSMB5	proteasome 20S subunit beta 5
scRNA-seq	single-cell RNA sequencing
TF	transcription factor
TAMs	tumor-associated macrophages
TCGA	The Cancer Genome Atlas
TIDE	Tumor Immune Dysfunction and Exclusion
TLSs	tertiary lymphoid structures
TNF	tumor necrosis factor
TSA	tyramide signal amplification
UCEC	uterine corpus endometrial carcinoma
UMAP	uniform manifold approximation and projection
