# Supplementary material for: Integrative Single-Cell and Spatial Transcriptomic Analysis Identifies a Tertiary Lymphoid Structure-Associated LAMP3+CCR7+ mregDC Antigen-Presentation Program in Ovarian Cancer
Source: Cancers (Basel). 2026 Jul 14;18(14):2259. doi: 10.3390/cancers18142259 (PMC13406827; doi:10.3390/cancers18142259)
Supplement: Supplementary file 1 [file cancers-18-02259-s001.zip › cancers-4402066-supplementary/Cancers_Proof_Final_Supplementary.pdf]

Supplementary information

Supplementary Figure Legends

Supplementary Figure S1. singleCellHaystack spatial non-randomness audit

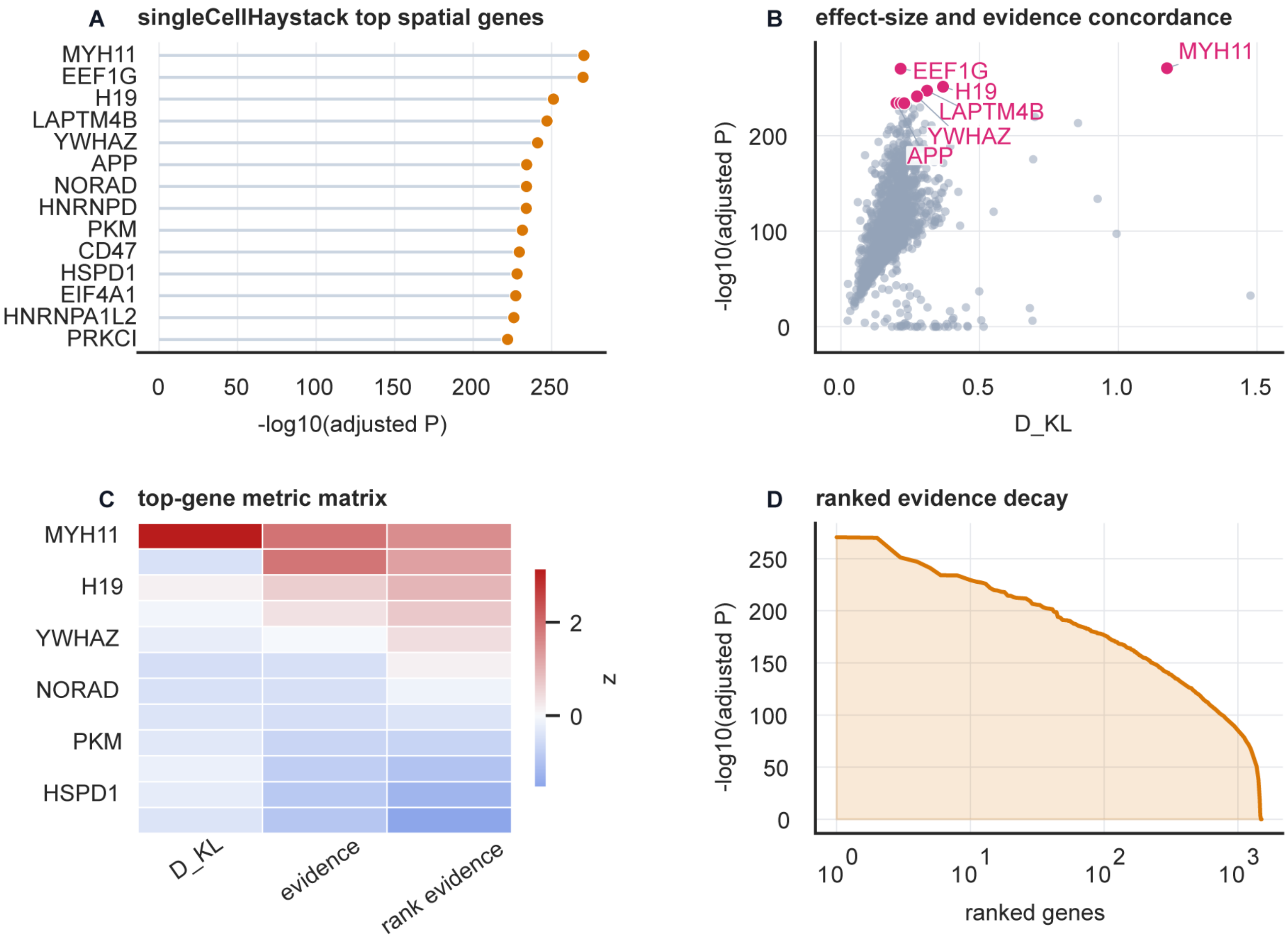

A, Top spatially non-random genes ranked by singleCellHaystack evidence in the Xenium-derived spatial subset. **B,** Concordance between D_KL and statistical evidence for the ranked gene set. **C,** Metric matrix for the highest-evidence genes, showing D_KL, nominal evidence, and adjusted evidence on a standardized scale. **D,** Ranked evidence-decay curve across the tested genes.

Supplementary Figure S2. Hotspot spatial-autocorrelation audit

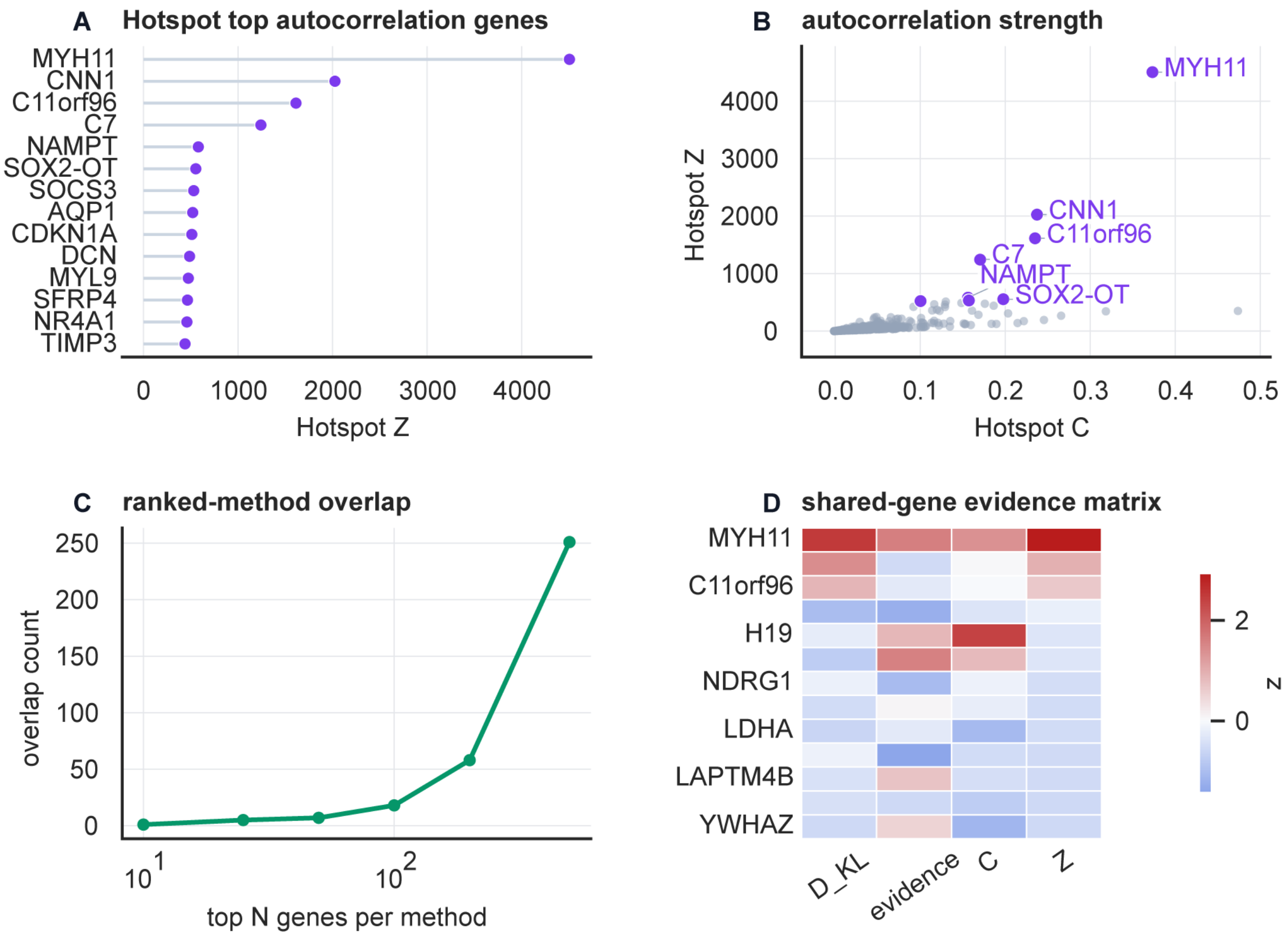

A, Top Hotspot spatial-autocorrelation genes ranked by Hotspot Z score. **B,** Concordance between Hotspot C and Z statistics. **C,** Overlap between Hotspot and singleCellHaystack prioritized genes across increasing rank thresholds. **D,** Shared-gene evidence matrix comparing singleCellHaystack and Hotspot statistics.

Supplementary Figure S3. Representative spatial core of the TLS/mregDC/AP-proxy niche

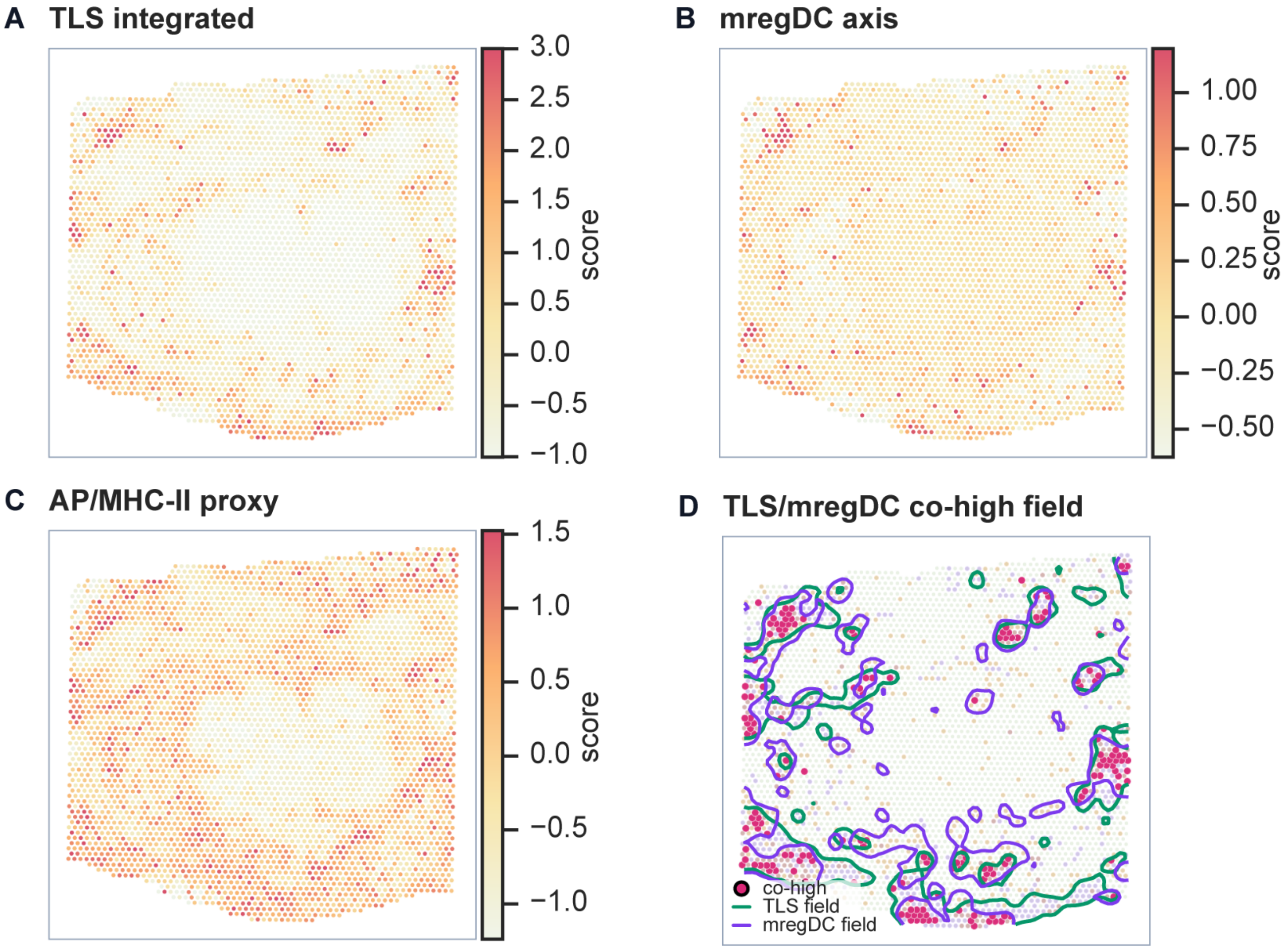

A, TLS integrated score map in a representative spatial sample. **B,** mregDC-axis score map in the same coordinate system. **C,** AP/MHC-II proxy score map. This score uses available antigen-presentation and MHC-II-associated genes and should not be interpreted as direct HLA-DRA measurement when exact HLA genes are absent from the panel. **D,** TLS/mregDC co-high cells overlaid with smoothed TLS-field and mregDC-field contours.

Supplementary Figure S4. Multi-sample spatial validation and gene-coverage audit

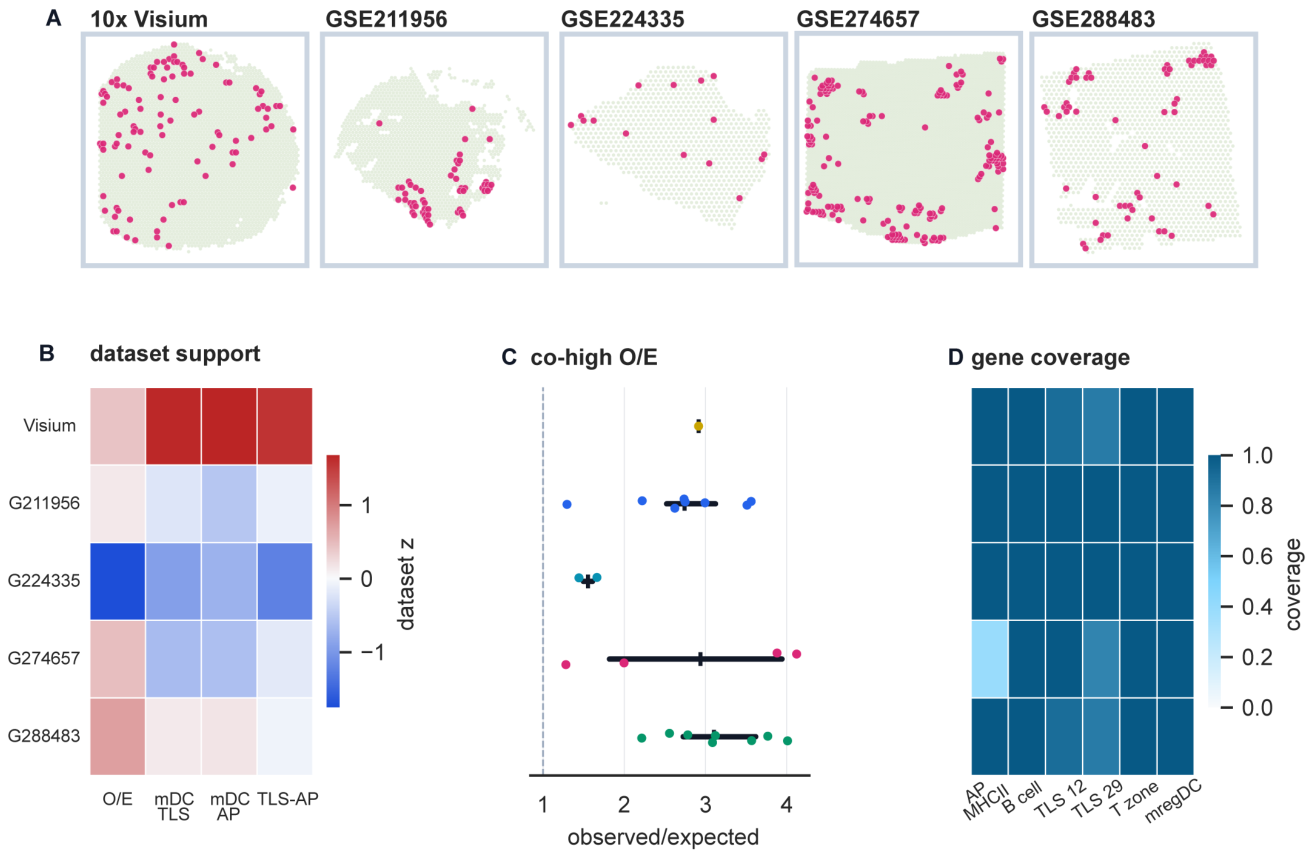

A, Multi-sample spatial overlays showing co-high spots or cells across public ovarian spatial-transcriptomic datasets. **B,** Dataset-level support for core spatial metrics, including observed-versus-expected TLS/mregDC co-high enrichment and mregDC/TLS/AP-proxy associations. **C,** Sample-level observed-versus-expected co-high enrichment distribution. **D,** Gene-coverage fraction for the spatial signatures used in the multi-sample analysis.

Supplementary Figure S5. Internal patient-level model validation

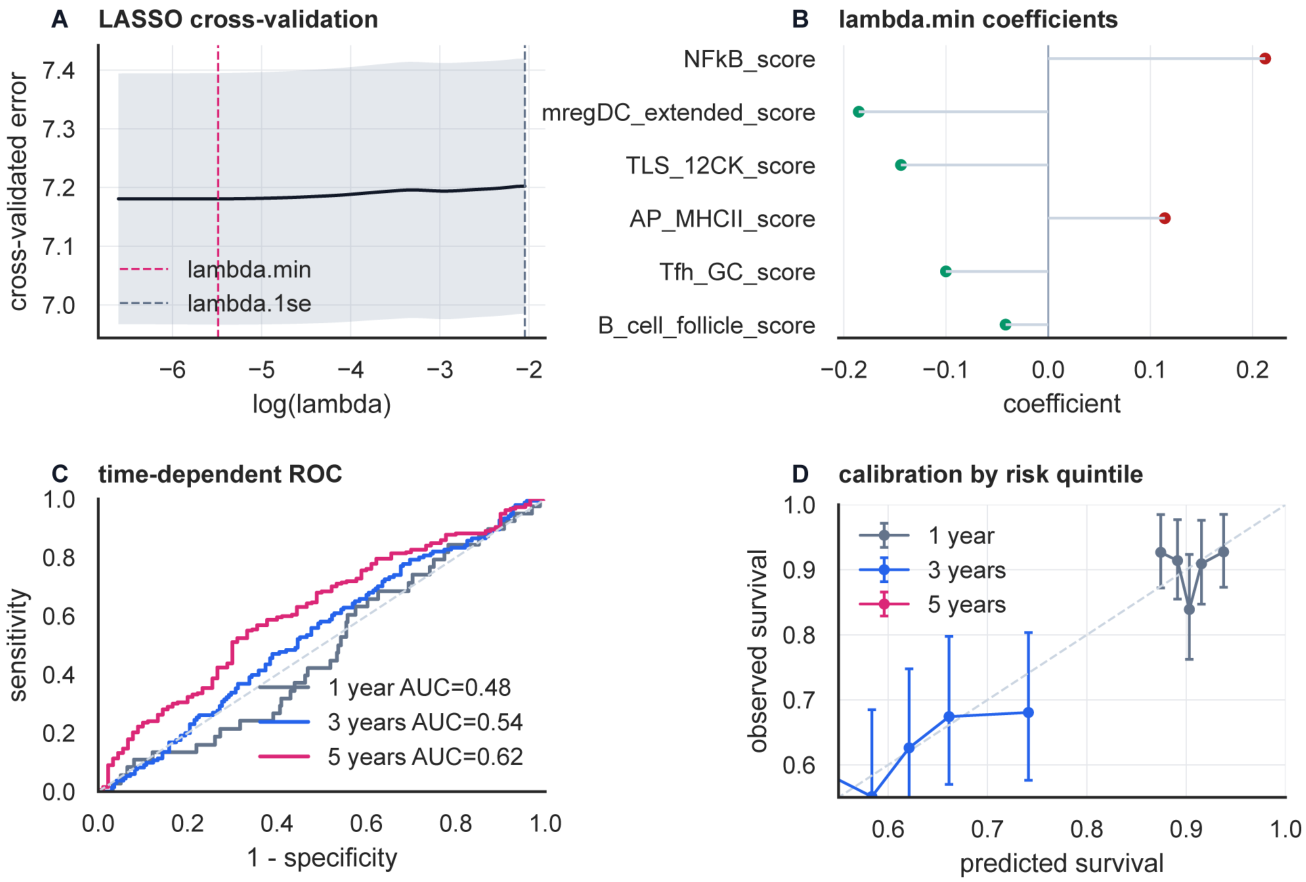

A, LASSO cross-validation curve with the selected penalty. **B,** Non-zero coefficients at the selected lambda value. **C,** Time-dependent ROC curves for 1-, 3-, and 5-year survival in the internal TCGA-OV assessment. **D,** Calibration by predicted-risk quintile. These analyses support internal model behavior and do not replace external clinical validation.

Supplementary Figure S6. Immune-context-adjusted specificity analysis

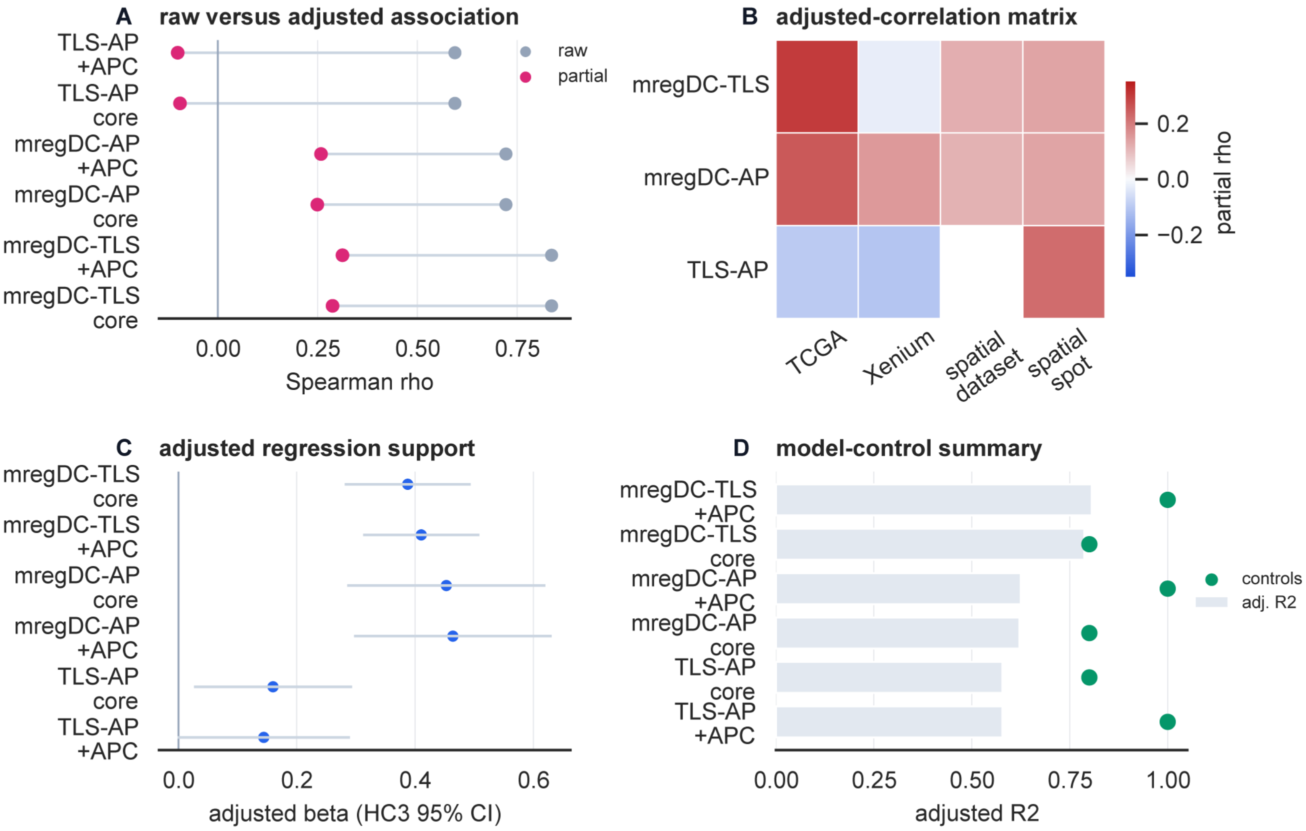

A, Raw versus partial correlations for mregDC–TLS, mregDC–AP/MHC-II, and TLS–AP/MHC-II relationships. **B,** Adjusted-correlation matrix after immune-context and lineage controls. **C,** Adjusted regression support for the same relationships. **D,** Control-set and model-summary panel. This audit is intended to show bounded specificity, not to claim complete independence from immune abundance.

Supplementary Figure S7. Exploratory drug-prioritization layer

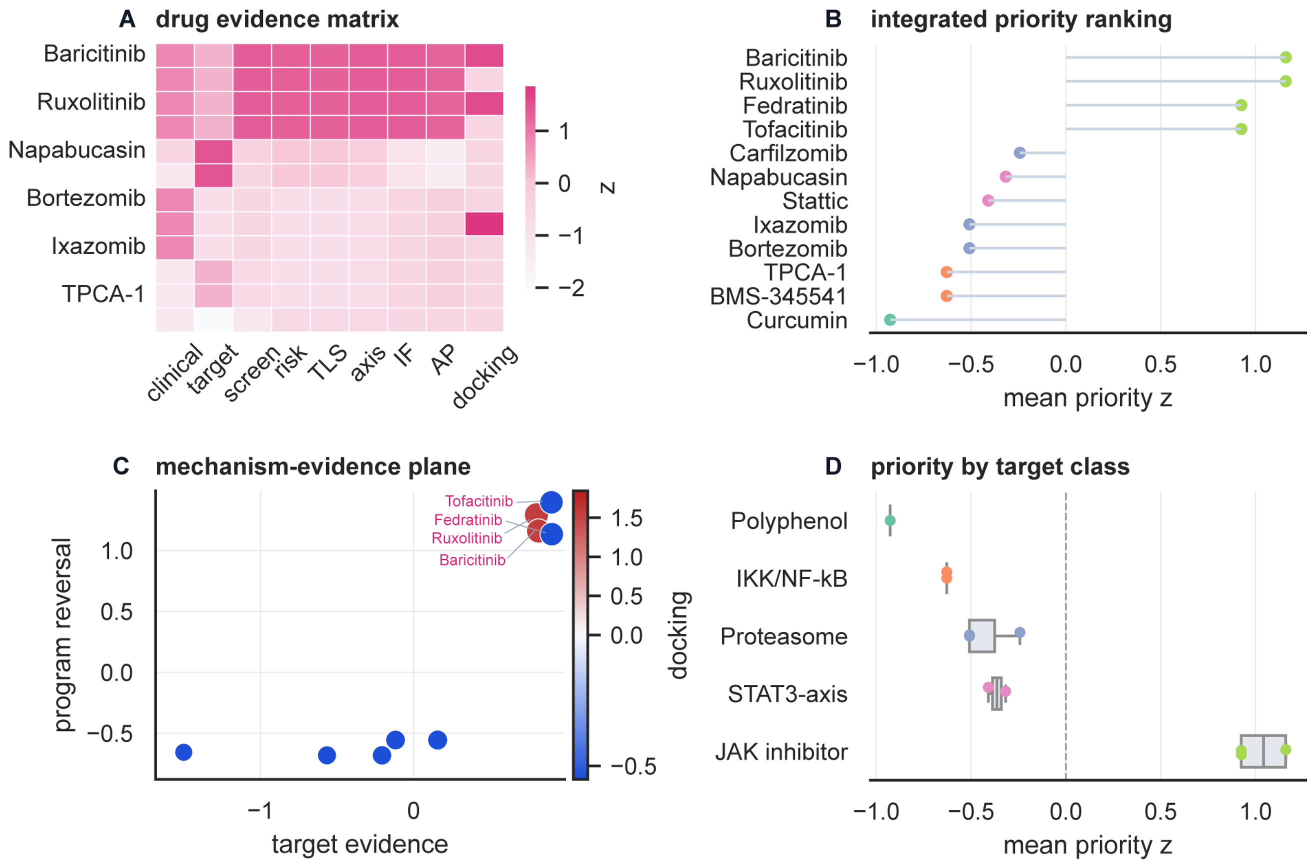

A, Compound-by-evidence matrix showing standardized support across target evidence, virtual screening, predicted program reversal, and docking layers. **B,** Integrated computational priority ranking. **C,** Relationship between target evidence and predicted program-reversal support. **D,** Priority distribution by target class. This figure is a hypothesis-generating computational screen and does not establish drug efficacy, pharmacologic response, or therapeutic applicability without experimental validation.

Supplementary Figure S8. scRNA-seq and representative IF support

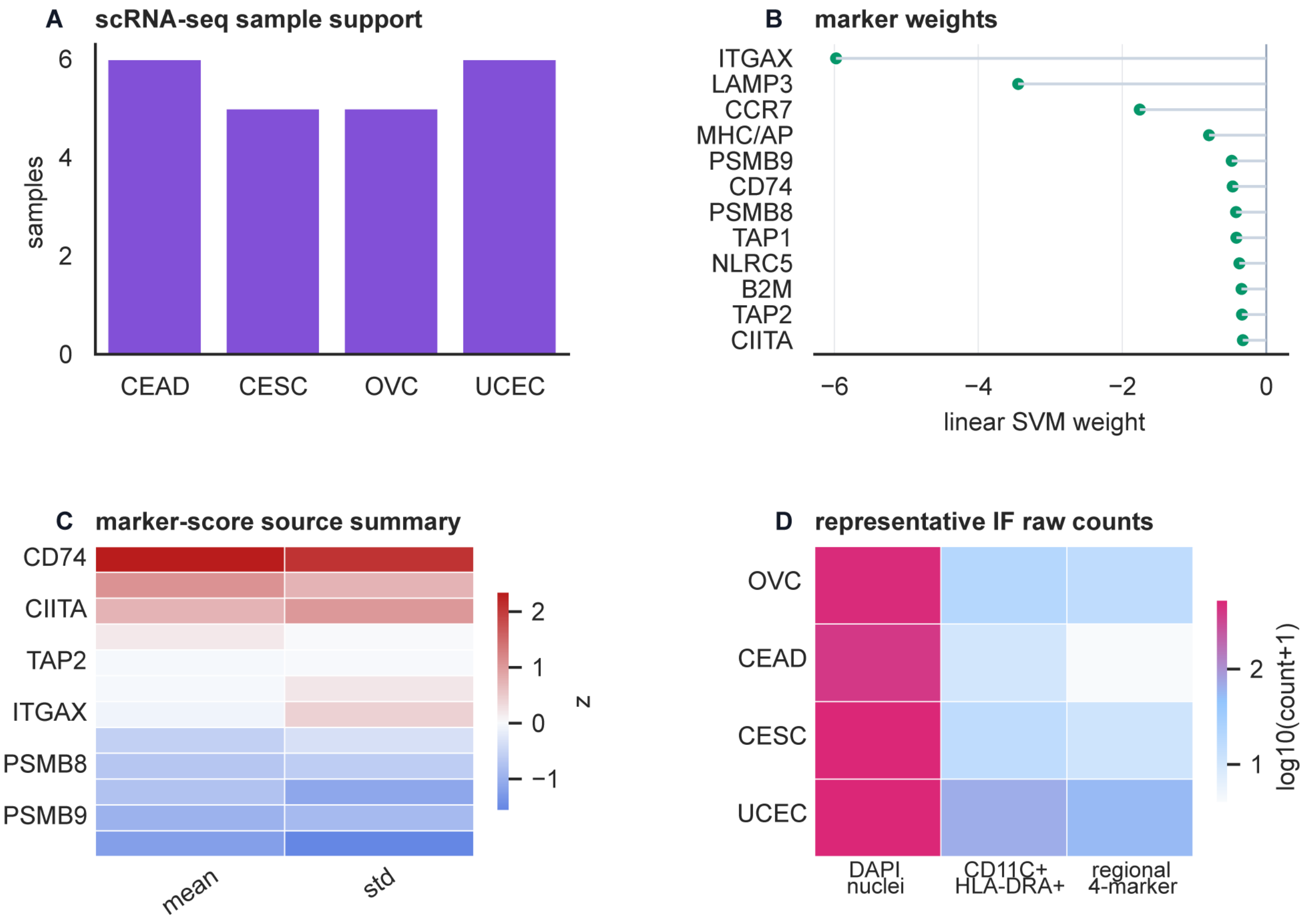

A, Disease and sample support in the scRNA-seq reference. **B,** Marker weights contributing to the mregDC/AP program. **C,** Marker-score source summary for selected mregDC and antigen-presentation genes. **D,** Representative IF raw count summary displayed as $\log_{10}$(count+1). The IF layer supports marker presence in representative gynecologic tumor images; it is not used to claim tissue-level cell density or separated-channel single-cell four-marker colocalization.

Supplementary Figure S9. Xenium program maps in a common spatial coordinate system

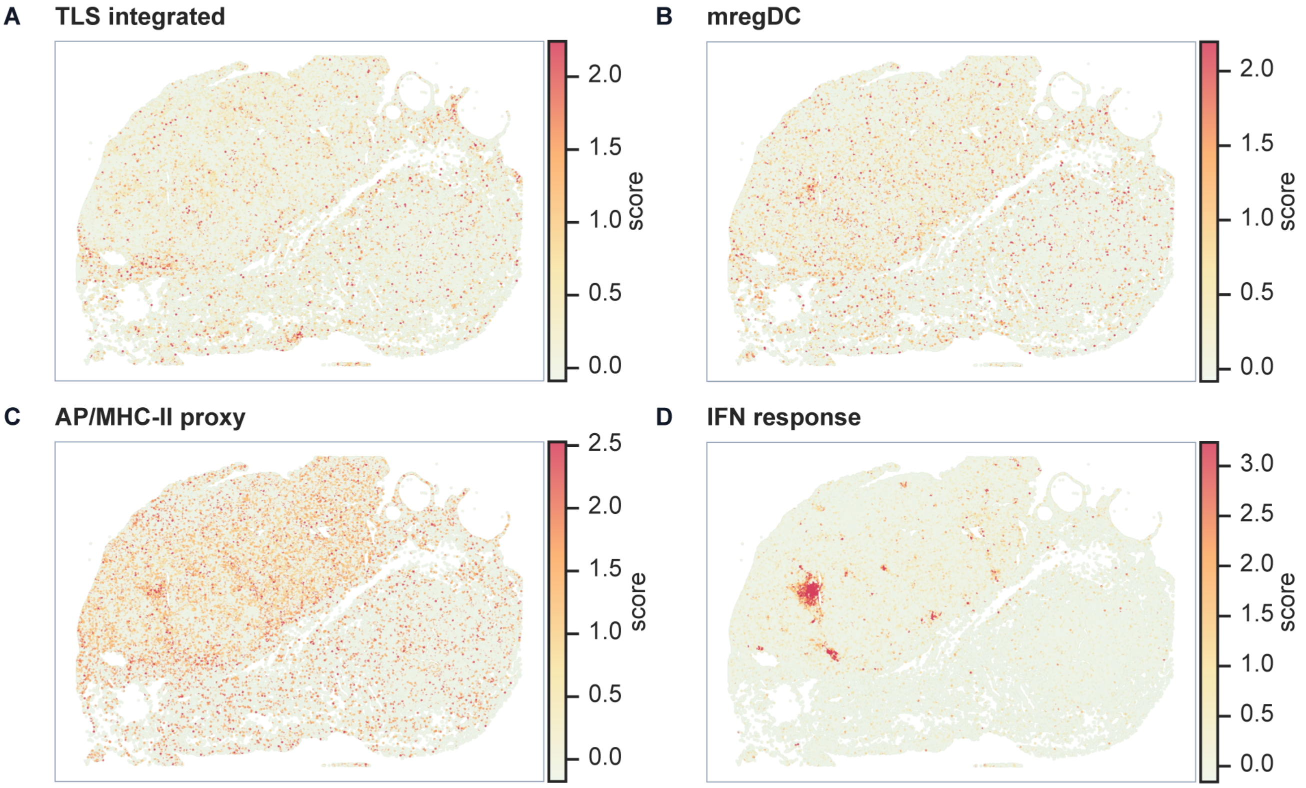

A, TLS integrated score map. **B,** mregDC program map. **C,** AP/MHC-II proxy map. **D,** IFN-response map. All panels use the same spatial coordinate system so that the relative spatial distribution of the programs can be compared.

Supplementary Figure S10. TLS-field segmentation and program coverage

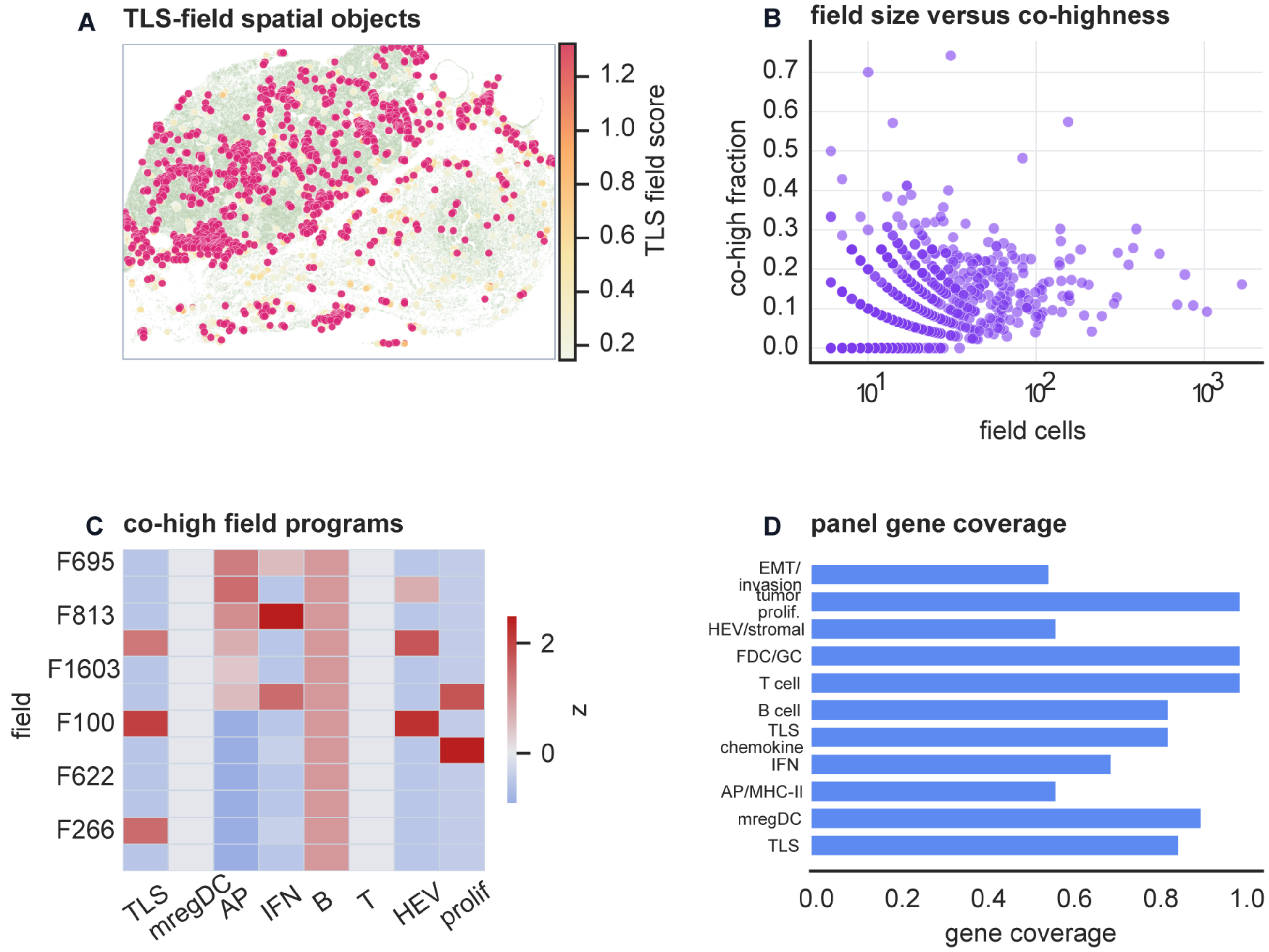

A, Tissue-background map with TLS-field grid objects and co-high bins highlighted. **B,** Field size versus TLS/mregDC co-high fraction. **C,** Program matrix across the highest co-high TLS fields; neutral tiles denote zero-centred or no-variance readouts rather than missing data. **D,** Xenium panel gene coverage for the programs used in the spatial interpretation.

Supplementary Figure S11. TLS distance and decile gradients

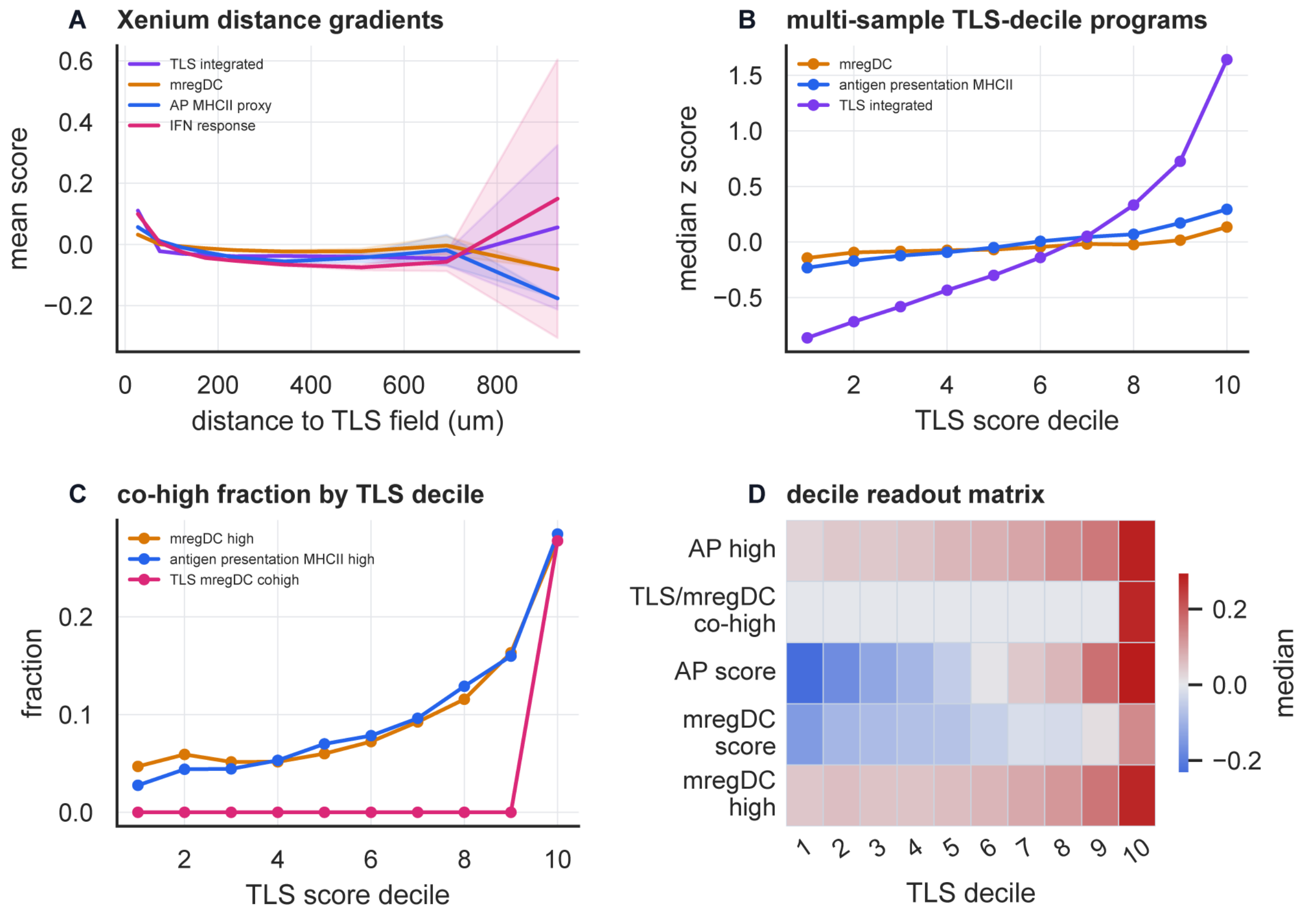

A, Xenium distance gradients from TLS fields for TLS, mregDC, AP/MHC-II proxy, and IFN programs. **B,** Multi-sample TLS-score decile trends for mregDC, antigen-presentation/MHC-II proxy, and TLS programs. **C,** Co-high fractions across TLS score deciles. **D,** Decile readout matrix for AP-high, mregDC-high, and TLS/mregDC co-high signals; neutral tiles denote zero or near-zero median readouts rather than missing data. Wider uncertainty at distant bins should be interpreted cautiously.

Supplementary Figure S12. Ten-method immune-deconvolution context

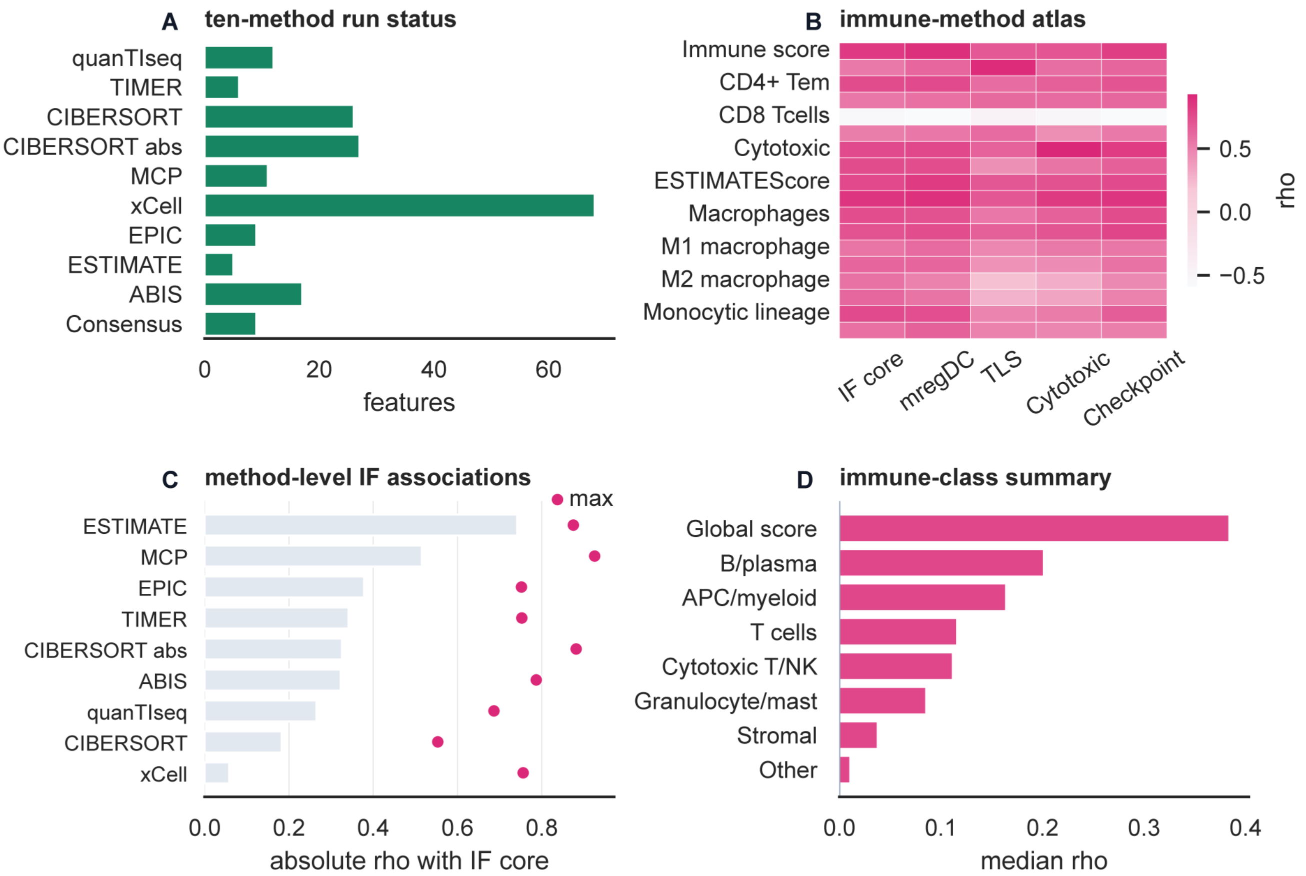

A, Status summary for the ten immune-deconvolution methods included in the final immune-context layer. **B,** Immune-method atlas showing associations between selected immune readouts and the TLS/mregDC axis. **C,** Method-level IF-association summary, showing median and maximum absolute associations. **D,** Immune-class summary across methods. Method heterogeneity is expected and is interpreted as context rather than perfect algorithmic agreement.

Supplementary Figure S13. Patient-level composite feature structure

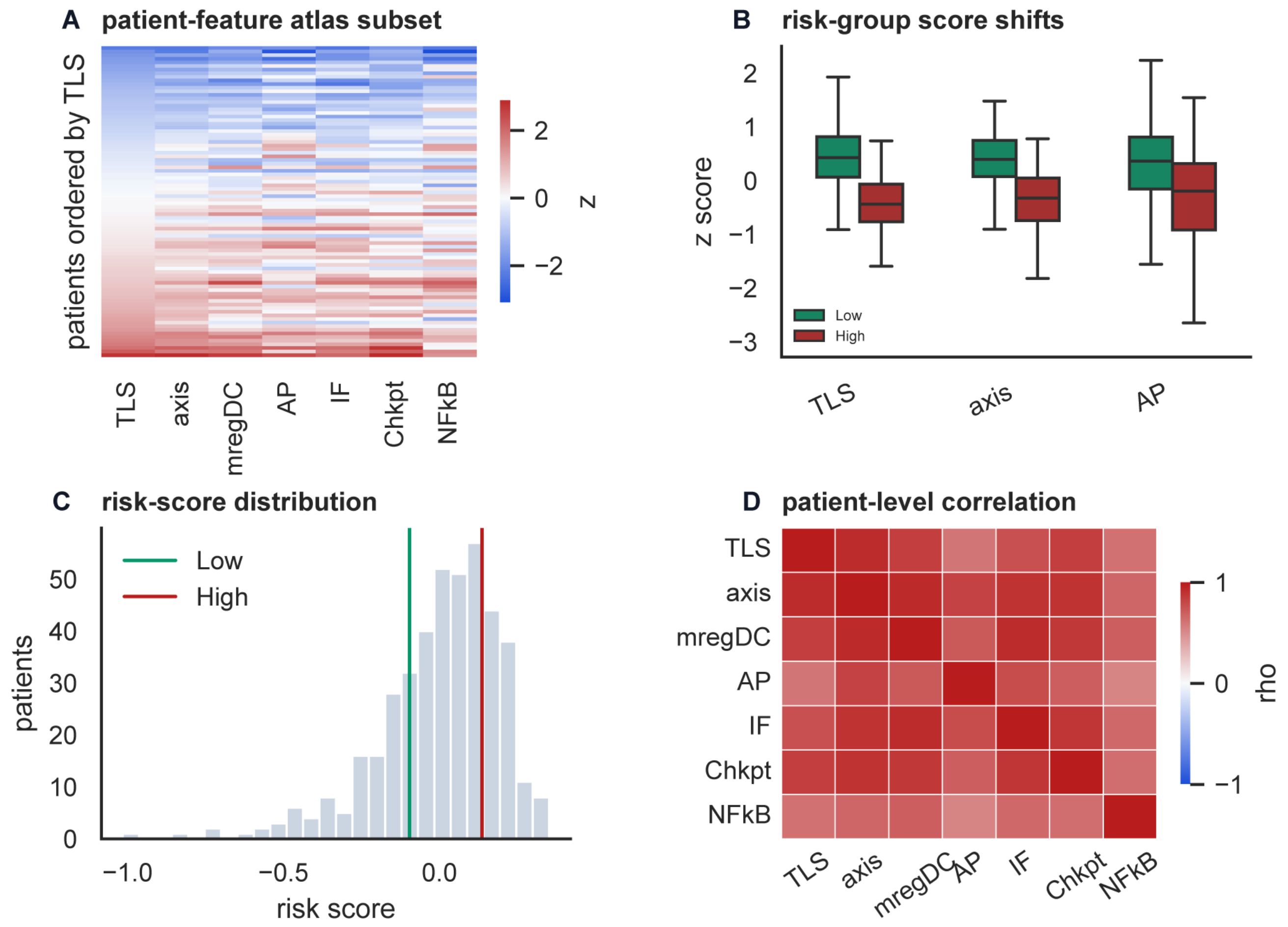

A, Patient-feature atlas subset for the TLS, mregDC, AP/MHC-II proxy, IFN, and NF-kB-related features. **B,** Risk-group shifts for selected program scores. **C,** Risk-score distribution by model-defined group. **D,** Patient-level correlation matrix across the composite features.

Supplementary Figure S14. Virtual perturbation support

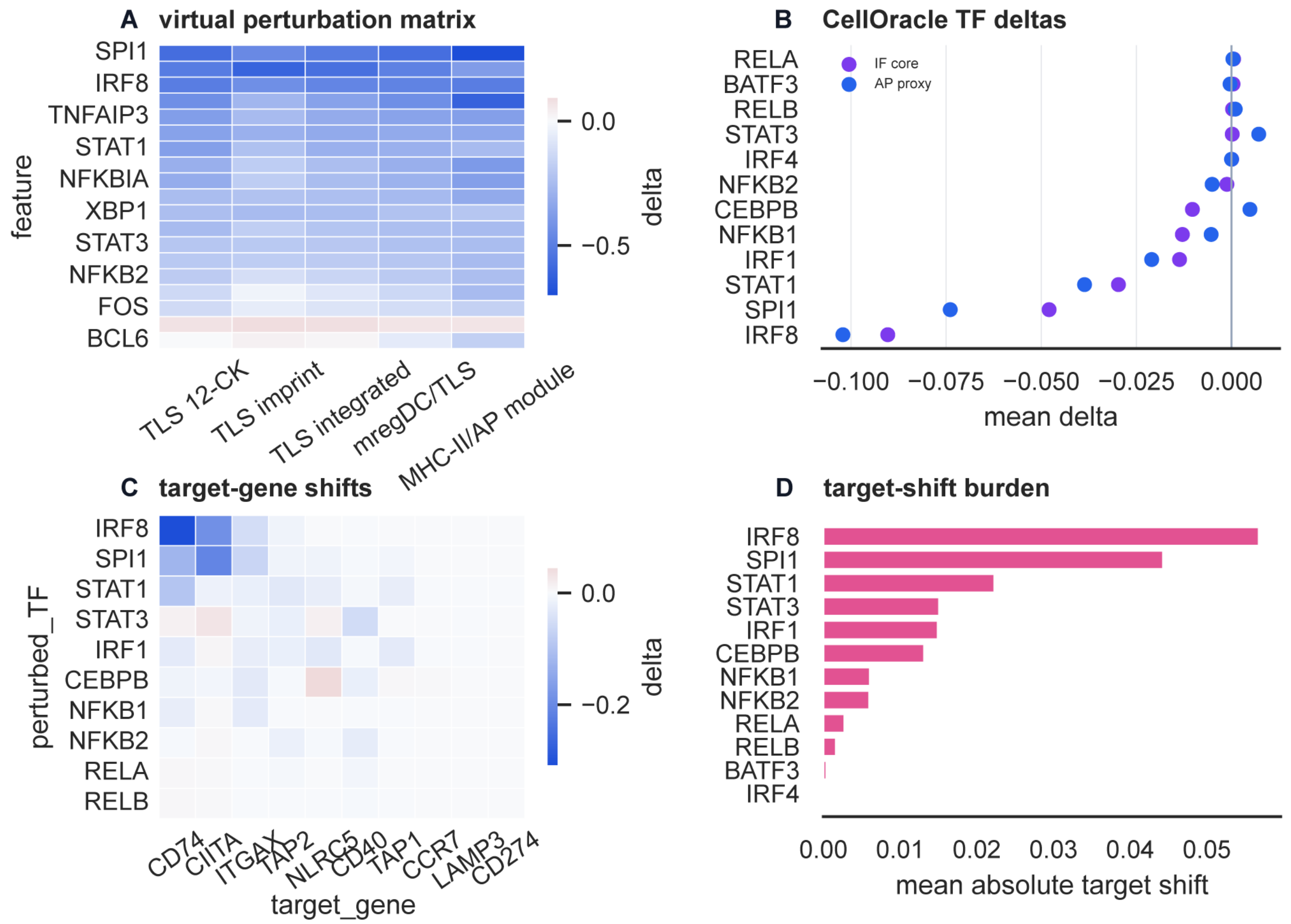

A, Virtual perturbation matrix across regulatory targets and programs. **B,** CellOracle-style TF delta summary. **C,** Target-gene shift matrix. **D,** Target-shift burden summary. These results are TF/program-level computational perturbation analyses and should not be interpreted as direct LAMP3 or CCR7 knockout experiments.

Supplementary Tables

Supplementary Table S1. Reconstructed 22-sample scRNA-seq GEO source manifest, disease labels, sample counts, and accession-level summaries.

Supplementary Table S2. Signature definitions, gene-coverage audits, marker-availability checks, and legacy-label harmonization for the mregDC, TLS, antigen-presentation/MHC-II, IFN, FDC/GC, HEV/stromal, proliferation, and EMT/invasion modules.

Supplementary Table S3. Public multi-sample spatial transcriptomic samples and public 10x/Xenium reference datasets used for spatial validation, including accession-level metadata and spot/cell counts where available.

Supplementary Source Data

- Source-data index: `source_data/SOURCE_DATA_INDEX.csv`.
- Fig. S1: `source_data/FigS01_singleCellHaystack_audit`.
- Fig. S2: `source_data/FigS02_Hotspot_audit`.
- Fig. S3: `source_data/FigS03_Xenium_spatial_maps` and `source_data/Fig04_Xenium_spatial_segmentation`.
- Fig. S4: `source_data/FigS04_multi_sample_spatial_source`.
- Fig. S5: `source_data/FigS05_model_validation` and `source_data/Fig09_LASSO_Cox_internal_validation`.
- Fig. S6: `source_data/FigS06_specificity_adjustment`.
- Fig. S7: `source_data/FigS07_drug_prioritization_docking`.
- Fig. S8: `source_data/Fig01_study_design_scRNA_reference`, `source_data/Fig02_scRNA_mregDC_program`, and `source_data/Fig03_representative_IF_raw_counts`.
- Fig. S9-S10: `source_data/Fig04_Xenium_spatial_segmentation`.
- Fig. S11: `source_data/Fig06_TLS_distance_gradient`.
- Fig. S12: `source_data/Fig07_ten_method_immune_deconvolution`.
- Fig. S13: `source_data/Fig08_patient_level_composite`.
- Fig. S14: `source_data/Fig10_virtual_perturbation`.
